# Exploring the ethical values and social drivers behind consumer preferences for cruelty-free products

**DOI:** 10.3389/fpsyg.2025.1660619

**Published:** 2026-01-29

**Authors:** Ebru Enginkaya, Munise Hayrun Sağlam

**Affiliations:** Department of Business Administration, Yıldız Technical University, Istanbul, Türkiye

**Keywords:** altruistic motivation, ethical consumption, corporate social responsibility, cruelty-free products, influencer advocacy, mixed-methods, price fairness, stimulus–organism–response (S–O–R)

## Abstract

**Introduction:**

Cruelty-free labels have moved from niche certification to mainstream expectation. Yet, little is known about how the multiple cues that accompany these products converge to turn moral intent into action. Addressing this gap, the present study reconceptualizes cruelty-free purchasing as a layered moral performance orchestrated by symbolic, social, and economic stimuli.

**Methods:**

A mixed-methods design combined a cross-sectional survey of 624 adult consumers framed within a Stimulus–Organism–Response (S–O–R) model with partial least squares structural equation modeling and 22 in-depth interviews, which were analyzed thematically.

**Results:**

Quantitative results show that the logo, influencer advocacy, and perceived corporate social responsibility image each elevate altruistic motivation (*β* = 0.282–0.539), which, together with ethical concern, explains 74% of the variance in cruelty-free buying. Price fairness moderates this pathway, such that motivation converts to purchase only when the premium is judged acceptable (interaction *β* = −0.15). Downstream, buying cruelty-free products strongly inspires self-expression (*β* = 0.843), social bonding (*β* = 0.745), and behavioral empowerment (*β* = 0.647). Qualitative themes, ranging from millisecond “ethical sparks” upon spotting the bunny icon to community-building rituals like #crueltyfreehaul, corroborate and enrich these statistical paths.

**Discussion:**

Together, the findings portray cruelty-free consumption as a script in which logos, parasocial voices, and fair prices jointly ignite compassion, channel it into purchase, and reward it with identity and community pay-offs. Practically, credible certification, authentic influencer partnerships, transparent corporate social responsibility communication, and fair-premium pricing emerge as levers for brands and policymakers seeking to translate compassion from intention to action across the expanding cruelty-free marketplace.

## 1 Introduction

Recent industry reports and academic analyses converge to show that the cruelty-free (CF) designation has shifted from niche certification to a mainstream purchasing criterion. Across bathroom shelves, kitchen cupboards, and even pet food aisles, the small rabbit emblem has leaped from obscurity to everyday shorthand for compassion. Industry surveys now reveal that nearly three out of four shoppers (73.9%) actively seek CF alternatives whenever possible (Amalia and Darmawan, 2023). The dollars have followed the sentiment: sales of animal-friendly household cleaners reached USD 6.97 billion in 2024 and are projected to grow at more than 11% CAGR through 2030 (Grand View Research, 2024a, 2024b; Statista, 2023). Likewise, CF personal care and beauty lines, which currently constitute a USD 6.2 billion market, are forecast to almost double by 2032 (Fact View Research, 2023). What unites these disparate categories is a shared moral promise: Ordinary consumption can spare non-human animals from harm. This expansion is closely intertwined with regulatory developments: in the European Union and the United Kingdom, comprehensive bans on animal testing for cosmetic products and most cosmetic ingredients, combined with marketing bans on newly animal-tested products, have effectively embedded CF positioning within the mainstream marketplace (European Commission, 2013; Cruelty Free International, 2023). In contrast, the United States has followed a more fragmented path, with a growing number of state-level “cruelty-free cosmetics” laws and ongoing federal debate over the Humane Cosmetics Act (Humane Society of the United States, 2017; Cruelty Free International, 2021). Several Asia–Pacific countries have likewise introduced bans or strong restrictions on cosmetic animal testing or, as in the case of China, relaxed mandatory testing requirements for many “general” products (Government of India, 2014; Eurogroup for Animals, 2019; RSPCA Australia, 2025; Premium Beauty News, 2021). Within this landscape, the Turkish market is not isolated but is continuously exposed to CF standards, labels, and narratives circulating through European regulation and multinational brand strategies, providing a relevant context for examining how ethical values and social drivers shape consumer preferences for CF products.

Despite this commercial momentum, scholarship still treats CF purchasing as a single-factor phenomenon, examining moral identity (Mouat et al., 2019) in one paper and price sensitivity (Curth et al., 2024) in another, leaving unanswered how multiple cues cooperate or compete at the point of choice. Moreover, prior quantitative studies often rely on Likert-type surveys, which are detached from the rapid, sometimes visceral heuristics shoppers deploy in real aisles (Bakr et al., 2023; Suphasomboon and Vassanadumrongdee, 2022), while qualitative work seldom anchors its insights in a formal explanatory model (Talavera and Sasse, 2019).

Parallel to this stream of work, recent studies on moral licensing further clarify how an initial “good” act shapes subsequent ethical consumption decisions. Wen and Hu (2023) show that moral licensing is not a fixed outcome. When individuals publicly share a prior moral behavior on social media, lower moral self-regard can *suspend* the typical licensing effect and increase the likelihood of engaging in another moral act, particularly in near-term decisions. Building on a broader spillover perspective, Gregersen et al. (2025) distinguish between positive spillover, negative spillover, and (moral) licensing, demonstrating that one ethical choice can either reinforce or undermine later behaviors depending on how it is embedded in social and contextual cues. In a pro-environmental setting, McCarthy (2024) finds that moral licensing can disrupt the standard path from perceived behavioral control to conservation behavior in solar households, as consumers vindicate wasteful energy use after an initial ethical investment, even though social influence remains a key driver of intentions. At the individual level, Shi and Xiao (2023) show that creativity can strengthen moral credentials and thereby increase unethical behavior, underscoring how identity-relevant traits interact with licensing dynamics. These findings portray moral licensing as a malleable, socially embedded process rather than a uniform effect. Within our S–O–R framework, they suggest that earlier cruelty-free choices and identity claims may either spill over into stable CF preferences or, conversely, license a return to conventional products when economic or social constraints are salient. This underscores the need to examine how ethical values, altruistic motivation, perceived consumer empowerment, and social drivers in the Turkish market can support more consistent CF purchasing rather than moral leniency.

Guided by the Stimulus–Organism–Response (S-O-R) paradigm, the present mixed-methods study brings that interplay into focus. We examine four stimuli that routinely co-occupy a CF package or smartphone screen: a certified logo, influencer advocacy, the brand’s perceived corporate social responsibility (PCSR) image, and price fairness (PF). We propose that these cues ignite altruistic motivation (AM) and sharpen ethical concern (EC), which in turn give rise to three response-level outcomes—purchase intention, identity expression, and behavioral empowerment.

Our work advances the field in three ways. First, by modelling symbolic, social, and economic cues simultaneously, we capture the trade-offs consumers face when a moral aspiration collides with a trusted brand name or an unfavorable price tag. Second, we integrate a large-sample PLS-SEM with 22 in-depth interviews, triangulating “cold” structural paths with “hot” narrative details—illustrating, for example, how a rabbit icon can trigger a split-second ethical spark that overrides habitual preferences. Third, we extend S-O-R research by tracing downstream psychological payoffs, self-expression, social bonding, and perceived self-efficacy, which are rarely examined in ethical consumption studies.

For this purpose, this article is organized as follows. Following the introduction, Section 2 develops the theoretical framework by reviewing the S-O-R paradigm (Hwang and Griffiths, 2017) in the context of CF consumption and detailing the symbolic (label), social (influencer), and economic (CSR and PF) stimuli. Section 3 presents the quantitative study, describing the sample, measurement scales, and PLS-SEM results. In Section 4, we outline the qualitative methodology and thematic analysis process, providing in-depth reporting of the seven themes derived from the interviews. Section 5 integrates the quantitative and qualitative findings, interprets them considering prior literature, and discusses the insights that emerge from their convergence. Section 6 summarizes theoretical contributions, practical implications, limitations, and avenues for future research. Finally, Section 7 concludes with overarching takeaways and offers targeted recommendations for both policy and marketing practice.

This study explores how PF and AM jointly influence CF purchasing behavior and its psychological consequences within the S–O–R framework. Despite extensive research on ethical consumption, little is known about how perceptions of fairness interact with moral motives to shape CF behavior, particularly in non-Western, middle-income contexts. Drawing on survey data from consumers in Türkiye, the study examines a moderated sequential mediation process linking PF and AM to CF buying behavior and three types of inspiration: SEI, BEI, and AI. The results identify PF as an economic boundary condition that enables moral intentions to translate into ethical purchasing.

The findings extend S–O–R-based models by conceptualizing PF and AM as interacting stimuli, enrich the organism component by distinguishing SEI, BEI, and AI as meaningful yet temporary outcomes of CF behavior, and contextualize these mechanisms within Türkiye’s socio-economic environment. In response to recent calls for theory-driven, mixed-methods insights into moral decision-making (Arman and Mark-Herbert, 2024; Sun, 2020), we reveal how packaging symbols, parasocial voices, and price tags co-script everyday ethics. We further argue that establishing such a ‘moral script’ is essential for brands and policymakers if compassion is to translate from intention to action across the expanding landscape of CF goods.

## 2 Study background

During the past 5 years, CF purchasing has emerged as a prominent research topic at the intersection of consumer psychology and sustainability studies. However, the evidence base remains fragmented across product categories, theories, and national settings. Table 1 condenses six recent SSCI-indexed studies that probe why consumers choose CF alternatives. Two broad observations emerge.

**Table 1:** Overview of SSCI-indexed empirical studies investigating antecedents of CFBB.

No.	Authors (year)	Context/sample /product focus	Method	Principal antecedents tested	Core insight
1	Grappe et al. (2021)	Québec, *n* = 450/CF cosmetics	Between-subjects experiment PLS-SEM	Claim credibility, animal-welfare concern, subjective norms	External (credibility) and internal (altruism, norms) jointly lift attitude → CF intention.
2	Bakr et al. (2023)	Canada vs. Kuwait, *n* = 617/Plant-based/CF meat	Survey PLS-SEM	Env-concern, CF motive, meat attachment, neophobia	CF motive boosts attitude; meat attachment suppresses it; TPB paths stable cross-culturally.
3	Schuitema and De Groot (2015)	UK/IE labs, *n* ≈ 200/CF shampoo and moisturiser	Factorial experiment	Price, brand, CF cue, carbon footprint	Green cues sway choice only if price low and brand familiar → CF premium limits uptake.
4	Amalia and Darmawan (2023)	Indonesia, *n* = 326/CF personal care	Survey PLS-SEM	Hedonism, env-value, knowledge, TPB	Attitude, PBC, norms predict intention; hedonism and env-value act via attitude.
5	Suphasomboon and Vassanadumrongdee (2022)	Thailand, *n* = 423/Green/CF cosmetics	Survey PLS-SEM	Functional, emotional, social value; EC	Functional value → EC → intention; EC strongest direct driver.
6	(Villena-Alarcón and Zarauza-Castro, 2024)	Spain, *n* = 300 + 5 influencers/CF beauty on Instagram	Mixed (content + survey)	Influencer credibility, hashtag use	Low hashtag usage; authenticity valued but CF uptake modest → message dilution on social media.

TPB, Theory of Planned Behavior; PBC, Perceived Behavioral Control.

First, symbolic and social signals, such as certified labels, altruistic concern, and perceived subjective norms, can enhance consumers’ attitudes and purchase intentions, but their influence is highly conditional. Research in Québec cosmetics (Grappe et al., 2021) and Indonesian personal care (Amalia and Darmawan, 2023) demonstrates that certified claims, altruistic concern, and subjective norms positively influence attitudes and intentions. However, Spanish Instagram research finds that when influencers post CF content only sporadically, uptake is modest despite high follower trust (Villena-Alarcón and Zarauza-Castro, 2024). Together, these studies suggest that logo credibility and influencer advocacy serve as necessary but insufficient triggers; they require consistent signaling and supportive context to translate into actual behavior.

Second, economic pragmatism often eclipses moral aspiration. A factorial experiment conducted in the United Kingdom and Ireland reveals that CF cues increase product selection only when price parity and brand familiarity are maintained; even modest price premiums undermine this effect (Schuitema and De Groot, 2015). A comparable dynamic is observed in the plant-based meat category, where CF motives elevate attitudes, but meat attachment and price heuristics suppress intention (Bakr et al., 2023). Extending this argument, Suphasomboon and Vassanadumrongdee (2022) demonstrate that functional value, rather than social admiration, most robustly predicts EC, which in turn drives purchasing behavior.

## 3 Theoretical background and hypothesis development

**Figure 1:**
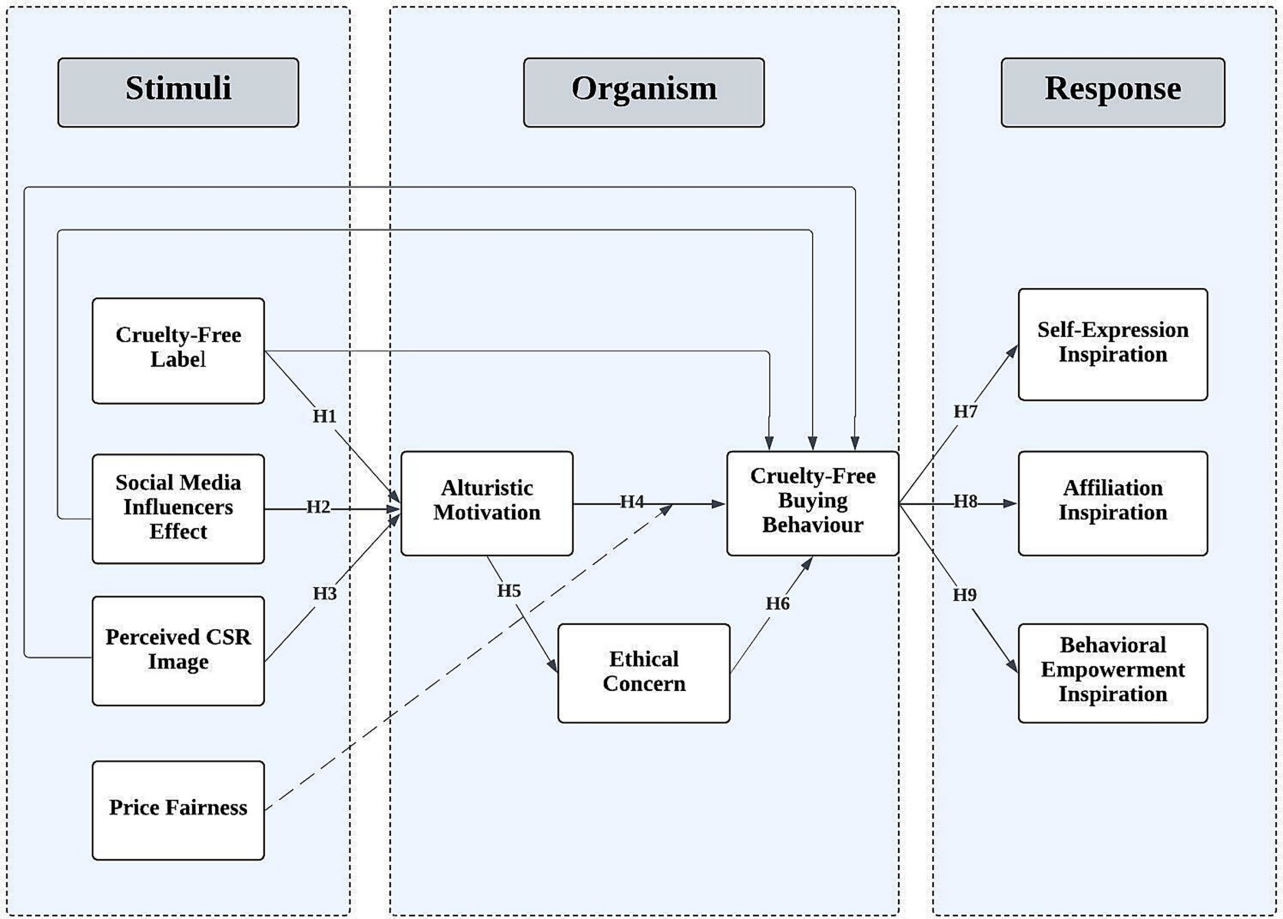
Conceptual framework grounded in the S-O-R paradigm.

Drawing on the S-O-R paradigm, we organize the literature review and hypothesis development around three layers. First, we examine marketplace stimuli that cue an ethical decision (certified CF label, influencer advocacy, PCSR image, and PF). Second, we discuss two organism-level mediators, AM and EC, that translate those cues into internal readiness to act. Third, we consider response-level outcomes comprising the focal behavior (CF buying) and its post-purchase psychological pay-offs (self-expression, affiliation, empowerment). Figure 1 depicts the resulting conceptual model.

### 3.1 Stimulus-level antecedents

#### 3.1.1 Cruelty-free label and altruistic motivation

CF labeling is an instantly recognizable moral cue, signaling that no animal suffered during the product’s development and appealing to consumers’ other-regarding concerns (Winders, 2006). Empirical research consistently demonstrates that this signal resonates with individuals who value animal welfare intrinsically. In cosmetics, for instance, respondents who notice a CF logo not only form more favorable attitudes but also report stronger purchase intentions, an effect that Wuisan and Februadi (2022) attribute to the importance that such consumers attach to safeguarding animals. Similar evidence is presented by Grappe et al. (2021), who demonstrate that CF logos harmonize ECs with self-presentation motives, yielding a distinctly positive affective response. (Munteanu and Pagalea, 2014; Lewis et al., 2016).

These findings accord with moral-heuristic theory: when shoppers face time or information constraints, they rely on easily processed “ethical shortcuts.” (De Pelsmacker et al., 2005). Sheehan and Lee (2014) show that a CF icon serves precisely this purpose, enabling quick alignment between the act of buying and the buyer’s moral identity. Such alignment is fundamentally altruistic because the primary beneficiary is a non-human other.

More broadly, sustainable consumption studies confirm that the desire to act in the interest of others—whether humans, animals, or the ecosystem—constitutes a pivotal motive behind ethical purchases (Waris et al., 2021; Schuitema and de Groot, 2015). Accordingly, a CF label should do more than enhance product appeal; it should kindle AM by reminding consumers that their choice can prevent harm to sentient beings.

*H1*. A CF label positively affects consumers' AM.

#### 3.1.2 Social media influencer advocacy and altruistic motivation

Influencers constitute a new class of value transmitters whose authenticity, narrative intimacy, and algorithm-enabled reach make them unusually effective at mobilizing other-regarding motives. Parasocial-relationship theory predicts that when a trusted content creator publicizes an ethical stance, followers are primed to adopt that stance as part of their own moral identity; recent work confirms the prediction across multiple contexts. For green cosmetics, social-media advocacy elevates subjective norms and, through them, AM to support CF brands (Pop et al., 2020). In a broader sustainability context, altruistic—as well as egoistic—motives mediate the effect of influencer messages on purchase intentions (Kumar and Pandey, 2023). Studies in high-credibility contexts show that trust in the messenger amplifies this moral contagion (Chavda and Chauhan, 2024).

The persuasive arc does not end with mere attitude change: altruistically motivated followers go on to circulate ethical content themselves, extending the influencer’s reach through peer sharing (Plume and Slade, 2018). Such network effects help explain why influencer campaigns are now a cornerstone of socially responsible branding strategies (Lokithasan et al., 2019). They also clarify why altruistic concern predicts online purchase intention for ethically positioned products, especially among digital-native cohorts (Traymbak et al., 2022). This evidence supports the proposition that influencer advocacy catalyzes AM, turning moral sentiment into market behavior.

*H2*. SMIs positively influences consumers’ AM.

#### 3.1.3 Perceived CSR image and altruistic motivation

CSR initiatives serve as moral signals that help consumers assess a firm’s alignment with their ethical standards (Sen and Bhattacharya, 2001; Chen and Kim, 2019). When the signal is interpreted as authentic and other-focused, the firm is reclassified from a market actor to a “moral in-group” partner, prompting a self-transcendent desire to advance shared prosocial aims—an archetypal form of AM (Bhattacharya and Sen, 2004). Crucially, this motivational lift hinges on perceived sincerity rather than the mere presence of a CSR program: high-fit, proactively communicated initiatives strengthen moral identification, whereas low-fit or transparently profit-driven efforts erode it (Becker-Olsen et al., 2005). Empirical work corroborates the mechanism across sectors: Consumers who ascribe altruistic motives to CSR in services and retailing report warmer brand evaluations and a stronger intention to reward the firm (Pérez and Bosque, 2015). Authentic, other-oriented messaging elevates moral emotions and measurably heightens AM (Wong and Dhanesh, 2017; Ji and Jan, 2019); influencer-mediated CSR endorsements likewise increase followers’ readiness to emulate prosocial behavior (Cheng et al., 2021). Meta-analytic evidence further shows that congruence between a consumer’s altruistic values and a focal CSR cause amplifies attitudinal and behavioral outcomes (Ribeiro et al., 2022). Together, these findings suggest that a credible CSR image operates as a psychological catalyst, converting ethical appreciation into an intrinsic willingness to act for the welfare of others.

*H3*. PCSR image positively influences consumers’ AM.

#### 3.1.4 Price fairness as boundary stimulus

PF sets the economic “permission structure” that determines whether moral motives can be enacted in the marketplace. Building on dual-entitlement theory, consumers simultaneously evaluate (a) their own right to a reference price and (b) the firm’s right to a reasonable profit (Kahneman et al., 1986). When a CF option is priced within this reference zone—or only slightly above it as a justifiable “moral premium”—the economic cost of acting ethically is perceived as tolerable, preserving the intrinsic warm-glow reward that follows prosocial choices (Curth et al., 2024). Equity-based appraisals then remain neutral or positive, allowing symbolic and social cues (such as a logo, influencer advocacy, or CSR) to reach the motivation stage unimpeded. If, however, the price breaches the entitlement boundary, the transaction is re-framed from “shared virtue” to “moral surcharge,” shifting attention from animal welfare to self-protection and heightening loss-aversion concerns (Bolton et al., 2003).

Crucially, fairness evaluations operate upstream, filtering how strongly AM translates into downstream cognitive and behavioral processes. Neuro-imaging work shows that prices deemed unjust extinguish reward-system activation triggered by prosocial cues long before a purchase decision is finalized (Chang et al., 2011). Survey and diary studies also reveal that consumers confronted with perceived overpricing quickly downgrade their moral intent to mere approval, preserving their self-concept while withholding action (Xia and Monroe, 2010; Chong et al., 2021; Maxwell, 2005). In short, PF functions as a boundary stimulus, not because it directly sparks or suppresses compassion, but because it modulates the pathway through which existing altruistic motives become concrete, CF purchases. This boundary role provides the conceptual scaffold for the moderated-mediation hypotheses (H13–H15) developed later in the paper without pre-empting their specific outcome-focused logic.

### 3.2 Organism-level mediators

#### 3.2.1 Altruistic motivation and cruelty-free buying

Altruistic motivation (AM)—defined as an internalized concern for the welfare of others—activates personal moral norms and a felt obligation to reduce harm (Stern et al., 1995). When such motivation is salient, CF labels become powerful moral affordances, enabling consumers to align marketplace choices with their prosocial values (Schuitema and de Groot, 2015; Magano et al., 2022). Experimental and survey evidence converge on this mechanism: psychosocial values that prioritize animal well-being enhance attitudes toward CF cosmetics and, in turn, purchase intentions (Grappe et al., 2021; Wuisan and Februadi, 2022). Longitudinal and cross-sectional studies further show that these attitudinal gains translate into behavior; altruistic concern predicts both stated intent and verified CF purchases across food, apparel, and beauty contexts (Jaiswal and Kant, 2018; Yazdanpanah and Forouzani, 2015; Prakash et al., 2024). Within the Theory of Planned Behavior, altruism serves as a core antecedent of pro-environmental attitudes (Huang et al., 2018), while value–belief–norm research suggests that moral obligation mediates the transition from intent to action (Chaudhary, 2018). Marketplace studies echo the finding that brands that embed altruistic cues in their positioning elicit stronger engagement and loyalty (Boccadoro et al., 2021). Additionally, social media communities amplify altruistic motives through peer endorsement and normative reinforcement (Kumar and Pandey, 2023). The evidence portrays altruistically motivated consumers as viewing CF products not merely as functional goods but as vehicles for enacting and reinforcing their ethical identity (Aggarwal et al., 2024), thereby increasing the likelihood—and persistence—of CF buying.

*H4*. AM positively influences consumers’ CFBB.

#### 3.2.2 Altruistic motivation and ethical concern

Altruistic orientations have a positive and significant impact on consumers’ attitudes toward ethical consumption (Oh and Yoon, 2014). This effect arises because individuals committed to altruistic values tend to internalize ethical consumption as part of their self-concept (Culiberg and Bajde, 2013; Chi, 2022; Bae and Yan, 2018). Even when deliberative processes obscure altruistic motives, intuitive, other-regarding reactions often yield ethically favorable decisions (Zhong, 2011). Accordingly, when consumers act on altruistic goals—such as reducing harm to animals or protecting vulnerable beings—their EC is likely to intensify (Le-Hoang, 2025; Chang and Chuang, 2020). They not only perceive cruelty-free options as preferable but also experience a stronger affective response to any potential moral transgressions (Schwartz, 1977). These insights lead us to the following hypothesis:

*H5. AM positively influences consumers’ ECs*.

#### 3.2.3 Ethical concern and cruelty-free buying behavior

Ethical concern (EC)—the cognitive-affective appraisal that one’s consumption choices carry moral consequences for sentient beings—acts as a pivotal catalyst in CFBB. Laboratory and field studies demonstrate that when empathy is considered in the decision-making process, consumers consistently prioritize products that prevent animal harm (Grappe et al., 2021; Michaelidou and Hassan, 2007; Fox and Ward, 2008). This effect is particularly pronounced among individuals with a pronounced internal locus of control, who are more likely to act when they believe their purchase can influence ethical outcomes (Lim et al., 2019; Dasunika and Gunathilake, 2021; Dissanayake, 2022). Experimental evidence further indicates that prompting moral reflection increases preference for ethically marketed goods (Peloza et al., 2013), while information on animal cruelty redirects demand toward CF options (Gunther et al., 2023). Collectively, these findings position EC as a behavioral driver rather than a mere attitude.

*H6*. EC positively influence consumers’ CFBB.

### 3.3 Response-level outcomes

#### 3.3.1 Cruelty-free buying behavior and self-expression inspiration

Choosing CF products enables consumers to establish a value alignment that extends beyond utilitarian exchange and serves as a symbolic self-presentation (White and Argo, 2011). Identity-based consumption theory posits an inspiration sequence: a brief “being inspired by” affective spark on recognizing a value match, followed by a “being inspired to” motivation to display that value publicly (Thrash and Elliot, 2003; Reed et al., 2012). In CF purchases, the act itself signals “I care about animal welfare,” supplying a potent “inspiration-by” trigger. Empirical evidence confirms that value-laden consumption catalyzes visual and behavioral self-expressions: admirers of ethical brands convert their purchases into identity markers that reinforce personal narratives and social images (Schau and Gilly, 2003; Schuitema and de Groot, 2015; van der Westhuizen and Kuhn, 2024). Likewise, Magano et al. (2022) demonstrate that altruism- and responsibility-based attitudes toward CF cosmetics encourage consumers to utilize their purchasing decisions as a platform for ethical advocacy. Together, these findings support the expectation that CFBB elicits SEI.

*H7*. CFBB positively influences SEI.

#### 3.3.2 Cruelty-free buying behavior and affiliation inspiration

Affiliation inspiration (AI) describes the brief but intense sense of “we-ness” that arises when an individual’s action resonates with a shared ethical norm or community identity (Schau et al., 2009). According to social identity theory, internalizing group values strengthens one’s sense of belonging (Bhattacharya and Sen, 2003). By choosing a CF product, consumers visibly signal membership in an “animal-friendly” community, igniting their AI. Moreover, generative cues—such as imagery emphasizing benefits for future generations—reinforce this communal bond by conveying that “our actions today serve the well-being of tomorrow’s members” (Ma and Xing, 2024). The resulting warm-glow effect further amplifies collective responsibility and the desire to unite with like-minded others (Tezer and Bodur, 2020). Taken together, these processes support the following hypothesis:

*H8*. CFBB positively influences AI.

#### 3.3.3 Cruelty-free buying behavior and empowerment inspiration

Psychological empowerment theory frames behavioral empowerment as the momentary surge of competence and agency that follows an act perceived as personally efficacious (Zimmerman, 1995). At its core are three mutually reinforcing routes—cognitive, emotional, and behavioral—through which an action nurtures intrinsic motivation (Thomas and Velthouse, 1990). CF purchasing activates each route. Cognitively, the shopper apprehends concrete animal welfare gains, imbuing the choice with meaning and personal relevance. Emotionally, the “warm-glow” effect accompanying prosocial action fortifies moral self-confidence (Chen et al., 2020). Behaviorally, enacting an ethical preference confirms one’s ability to effect change, thereby elevating self-efficacy and encouraging sustainable decisions in the future (Bandura, 1997). Empirical work shows that such empowerment experiences arise whenever technology or information grants users new capacities, whether through e-government portals (Li and Gregor, 2011) or peer-generated reviews that expand choice autonomy (Hu and Krishen, 2019). Accordingly, CF consumption should reliably kindle BEI by informing, energizing, and mobilizing consumers in a single, self-reinforcing episode.

*H9* CFBB positively influences BEI.

### 3.4 Sequential mediations

The CF logo operates as a high-diagnostic moral heuristic: its instantly recognizable imagery short-circuits elaborate information processing and evokes an automatic empathic response toward potential animal victims (De Pelsmacker et al., 2005; Sheehan and Lee, 2014; Greenwald et al., 2002). Neuroimaging research shows that such “no-harm” symbols activate the anterior insula and other regions associated with moral emotion, thereby foregrounding prosocial values at the point of choice (Feng et al., 2022). When this affective trigger aligns with a consumer’s self-schema, it intensifies social–moral identity salience and elevates AM—the felt obligation to act for others’ welfare (Connelly et al., 2011; Park and Lin, 2020a, 2020b; Shaw et al., 2016).

AM then feeds the value–belief–norm (VBN) sequence by converting empathic concern into an internalized sense of moral duty (Stern, 2000). Meta-analytic evidence across ethical-consumption domains confirms that AM is the most potent precursor of EC and that concern, in turn, predicts both stated and revealed purchasing after controlling for price and quality perceptions (Schamp et al., 2023; Fan et al., 2022). EC functions as a cognitive dissonance regulator: acting on it preserves moral self-consistency and thus lowers the psychological cost of paying a potential price premium (Schwartz, 1977; Griskevicius and Tybur, 2010). Field experiments corroborate the chain: removing the CF logo diminishes AM, suppresses ECs, and cuts CF sales by up to 30% (Tully and Winer, 2014).

*H10*. The presence of a certified cruelty-free label (CFL) increases CFBB via the sequential mediators AM and EC.

Social media influencers (SMIs) translate personal ethics into public scripts that combine the intimacy of peer talk with the reach of mass communication. Their strategic self-disclosure and perceived authenticity create parasocial bonds that prime followers to accept the influencer as a credible moral model (De Veirman et al., 2017). The social-cognitive theories propose that observing such a model triggers vicarious learning, whereby individuals internalize both the desirability and the efficacy of the behavior being modeled (Bandura, 1986). Experimental work corroborates the mechanism: value-congruent influencer endorsements significantly raise AM—measured as willingness to sacrifice personal gain for animal welfare—and this motivational lift mediates the effect on purchase intent for CF products (Lou and Yuan, 2019). Large-sample survey evidence supports the finding that perceived influencer authenticity elicits moral elevation, which in turn predicts intentions to adopt sustainable goods (Ki et al., 2020). A recent meta-analysis of cause-related marketing confirms that communicator–cause value fit systematically amplifies both attitudinal and behavioral outcomes across 85 independent samples (Fan et al., 2022). Once aroused, AM feeds the value–belief–norm cascade by crystallizing into EC (Stern, 2000). This proximal cognitive driver aligns behavior with moral self-standards, thereby facilitating CF purchasing (Schwartz, 1977). Collectively, the evidence supports a serial pathway in which influencer advocacy heightens AM, which then solidifies into EC, ultimately propelling CF buying.

*H11*. SMI advocacy increases CFBB through the sequential mediators AM and EC.

A persuasive CSR track record can do more than burnish a brand’s reputation—it offers consumers a concrete proof point that their own moral compass and the firm’s ethical stance are pointing in the same direction. Signaling theory shows that such “other-regarding” cues cut through marketplace noise by conveying costly commitment; social-identity research further demonstrates that consumers readily absorb committed firms into their moral in-group, experiencing the company’s prosocial aims as personally relevant (Bhattacharya and Sen, 2004). This perceived value match sparks AM, a motivational state that expands the self to include vulnerable out-groups such as laboratory animals. In the value–belief–norm cascade, AM solidifies into EC—the cognitive conviction that sparing animal suffering is a non-negotiable moral duty (Stern, 2000). Empirical work confirms the chain: longitudinal panel data reveal that heightened EC predicts not only stated intentions but also verified spending on CF cosmetics even after controlling for price sensitivity and brand familiarity (Pérez and Bosque, 2015). Neuroeconomic studies provide convergent validity, demonstrating that CSR endorsements consistent with a consumer’s core values increase activity in brain regions associated with altruistic reward, which in turn predicts actual purchasing (Lee et al., 2021). Acting on that concern protects moral self-integrity, nudging consumers toward CF options when trade-offs arise (Schwartz, 1977).

*H12*. PCSR image increases CFBB by sequentially elevating AM and then EC.

### 3.5 Moderated indirect effects

Price perceptions set the economic stage on which moral motives play out. When a CF option is priced within a range consumers deem “fair,” the cognitive cost of acting on altruistic motives collapses, allowing those motives to steer choice (Xia et al., 2004; Habel et al., 2016). Fair prices signal that the firm is not monetizing empathy, thereby sustaining the warm-glow reward that typically follows prosocial action (Tully and Winer, 2014; Jeong, 2024). Laboratory evidence shows that even highly other-regarding consumers curtail ethical purchases once they sense exploitative mark-ups, whereas price-parity scenarios nearly double uptake (Schuitema and De Groot, 2015). Field experiments in green retailing replicate this pattern: AM predicts actual basket share only when perceived PF is present; when fairness is questioned, moral intent stalls at the attitudinal stage (Haws et al., 2014).

Consumers who perceive prices as fair and choose animal-friendly brands grounded in ethical values simultaneously nourish both altruistic (societal benefit–oriented) and self-expressive motivations (Kennedy and Kapitan, 2022; Hamilton et al., 2020). Such choices enable individuals to articulate and signal their identities through ethical behavior (Achar et al., 2025). When altruistic motivations converge with a propensity to purchase cruelty-free products, ethical consumption acquires personal significance and becomes a vehicle for self-representation (Rangel-Lyne et al., 2021). Moreover, prior research shows that perceptions of price fairness enhance consumer trust, thereby reinforcing this process and deepening the self-expressive dimension of ethical consumption (Li et al., 2025; Heidary and Pluut, 2025).

Once the price hurdle is cleared and the CF product is bought, the act becomes a tangible artefact for identity work. Moral identity theory argues that enacting a cherished value in public space generates “symbolic self-completion,” a surge of SEI that invites consumers to display who they are and what they stand for (Aquino and Reed II., 2002; White and Argo, 2011). Purchases that spare animals from harm are especially potent self-signals because they merge compassionate intent with visible marketplace behavior (Berger and Heath, 2007). Neuroaffective studies confirm that fair-priced, ethical choices activate reward circuits linked to self-relevant meaning, whereas overpriced “ethical luxuries” trigger counterfactual regret and dampen identity expression (Pombo and Velasco, 2021).

*H13*. The interaction of PF and AM enhances SEI through the mediating role of CF buying behavior.

The consumer-empowerment theory argues that perceiving one’s action as efficacious and morally meaningful triggers a short-lived yet intense surge of behavioral empowerment—an affective state that reinforces future self-directed change (Zimmerman, 1995; Thomas and Velthouse, 1990). When AM is already high, a fair price acts as a catalytic cue, confirming that the firm is not exploiting consumers’ compassion and thereby sustaining the intrinsic “warm-glow” reward associated with prosocial choice (White and Peloza, 2009). Under such conditions, the CF purchase functions as a concrete micro-arena for exercising agency: consumers see their choice as a deliberate intervention in the marketplace rather than a passive response to marketing stimuli. This sense of “voting with one’s wallet” not only validates existing altruistic motives (Shaw et al., 2006; Moraes et al., 2011; Papaoikonomou and Alarcón, 2017) but also encourages consumers to generalize their perceived efficacy to future decisions in adjacent ethical domains, thereby laying the groundwork for more durable BEI.

Neuro-affective evidence suggests that fair-priced, ethical purchases activate ventral-striatal circuits linked to agency; however, identical products priced at a perceived surcharge blunt this signal and suppress post-purchase empowerment (Granato et al., 2022). Field data from sustainable apparel further indicate that only when PF is perceived do altruistically motivated consumers report a heightened sense of control and intention to leverage their buying power for broader social causes (Habel et al., 2020). In summary, PF amplifies the translation of AM into a CF purchase, and that purchase, in turn, inspires behavioral empowerment.

*H14*. The interaction of PF and AM enhances BEI through the mediating role of CFBB.

AI arises when an action visibly aligns the self with a valued moral community, producing a brief but potent “we-ness” sensation (Bhattacharya and Sen, 2003). A fair price makes this communal signal possible: it reassures altruistically inclined shoppers that joining the CF community does not entail economic exploitation, thereby preserving the social legitimacy of the act (Xia et al., 2004). Beyond this diffuse sense of belonging, affiliation-based inspiration is strengthened when consumers see that their CF choices are noticed, discussed, and endorsed by significant others. Research on ethical consumers and social customer journeys shows that shared experiences, conversations, and co-consumption episodes turn individual ethical purchases into relational events that affirm group membership and mutual commitment to moral goals (Hamilton et al., 2020; Kennedy and Kapitan, 2022). In settings where moral communities or cause-based groups are explicitly signaled, ethical labels can even invite consumers to align themselves with broader solidarity movements and to express support for stigmatized or underserved communities (Achar et al., 2025). Warm-glow studies show that fair-priced, ethical choices evoke stronger feelings of social connectedness than overpriced counterparts, even when the objective savings are identical (Tezer and Bodur, 2020). Large-scale survey evidence from plant-based food markets confirms that PF moderates the link between altruism and purchase; only under fair-price conditions does the purchase elevate perceived group identity and peer approval (Jia et al., 2023). Thus, fair pricing functions as a boundary condition that allows altruistic motives to materialize in a CF purchase, thereby sparking inspiration for affiliation.

*H15*. The interaction of PF and AM enhances AI through the mediating role of CF purchasing behavior.

## 4 Materials and methods

Given the study’s objective to estimate a multi-construct S–O–R model that includes an interaction term (PF × AM), multiple indirect paths, and three distinct post-purchase inspiration outcomes (SEI, BEI, AI), we employ an explanatory mixed-methods design. The quantitative phase tests the complete nomological network and the moderated-sequential mediation structure using PLS-SEM, which is well-suited for prediction-oriented modeling with multiple latent constructs and for assessing interactions and indirect effects via bootstrapping. The qualitative phase complements these tests by eliciting consumers’ interpretations of CF cues and price (un)fairness in real purchase narratives, thereby clarifying mechanisms, surfacing boundary conditions (e.g., scepticism toward claims or influencers), and strengthening interpretive validity. Together, the two phases provide a coherent justification for the methodological approach and enable triangulation of statistical patterns with lived accounts of CF decision-making.

### 4.1 Quantitative strand: structural model results

#### 4.1.1 Participants and sampling

A cross-sectional sample of N = 624 adult consumers residing in Türkiye was recruited via a professional online panel provider. We employed a stratified quota sampling strategy to ensure proportional representation across key demographic strata: gender (52% female, 48% male), age (18–24 = 18%, 25–34 = 37%, 35–44 = 29%, 45 + = 16%), and NUTS-1 region (Marmara, Central Anatolia, Aegean, Mediterranean, Black Sea, Eastern, and Southeastern Anatolia). This approach was chosen because purchase motivations, price sensitivity, and social influence cues are known to vary systematically by demographic segment. Stratification reduces sampling error, while quota controls mitigate overrepresentation, thereby enhancing the generalizability of findings to Türkiye’s adult population (Dillman et al., 2014).

Participants were eligible if they (a) purchased personal-care or household products at least once per month and (b) used a smartphone for shopping-related activities—criteria selected to reflect real-life exposure to CF product cues. An *a priori* power analysis using G*Power 3.1 (f^2^ = 0.02, *α* = 0.05, power = 0.95, 10 predictors) indicated a minimum required sample size of 474; the final sample comfortably exceeded this threshold. Of 721 invitations distributed, 654 responses were received (90.7% response rate), and 30 were excluded due to inattentive responding or survey completion times below one-third of the median, resulting in 624 valid responses. As shown in Table 2, sample demographics closely mirror national census figures, further supporting the external validity of the dataset.

**Table 2:** Socio-demographic profile of the sample.

Variable	Category	*n*	%
Gender	Female	324	52
	Male	300	48
Age group	18–24	112	17.9
	25–34	231	37
	35–44	181	29
	45 +	100	16
Education	High-school or lower	169	27.1
	Associate/Bachelor’s	306	49
	Master’s/PhD	149	23.9
Occupation	Student	48	7.7
	Public sector employee	181	29
	Private sector employee	237	38
	Self-employed/entrepreneur	51	8.2
	Academic/researcher	73	11.7
	Not currently employed	34	5.4
Monthly household income	< 22,150	68	10.9
	22,150–50,000	134	21.5
	50,001–80,000	143	22.9
	80,001–110,000	110	17.6
	110,001–150,000	87	13.9
	> 150,001	82	13.1

Sample size: *N* = 624.

#### 4.1.2 Measures and instrument development

All latent constructs were operationalized with multi-item, reflective scales adapted from prior peer-reviewed research. Scale wording was first adjusted to the CF products context, then subjected to a double back-translation procedure (English ↔ Turkish) to secure semantic equivalence. A panel of three marketing scholars and two industry practitioners evaluated the content validity, resulting in minor lexical refinement.

Items for SEI and AI (3 each) were adapted from Schau et al. (2009) and Venn et al. (2017). AM (4 items) was drawn from Goldsmith et al. (2000) and Prakash et al. (2024). The eco-label (CF logo) perception scale (3 items) followed by Nittala (2014) and Song et al. (2020). EC (4 items) relied on Suphasomboon and Vassanadumrongdee (2022). The PCSR image (7 items) was adapted from Achabou and Ho’s environmental CSR scale and tailored to the CF domain (Achabou, 2020; Ho, 2017; Huang et al., 2022). PF (6 items) combined wording from Petrick (2002), Martin et al. (2009), and Chung and Petrick (2013). The five-item SMI credibility scale was customised by De Veirman et al. (2017). CFBB (6 items) was adapted from Khare (2015), and behavioral-empowerment inspiration (3 items) from Speer and Peterson (2000), Christens (2012), and Li et al. (2021). The original scale items are listed in Appendix Table A.

All items were anchored on a seven-point Likert scale (1 = “strongly disagree” to 7 = “strongly agree”). A pilot test with 80 respondents confirmed readability and yielded satisfactory internal consistency reliabilities (Cronbach’s α ≥ 0.78). Pilot data also showed no cross-loading above 0.30 in an exploratory factor analysis, supporting preliminary discriminant validity. Final measurement properties (indicator loadings, AVE, CR, α) are reported in Table 3 and meet recommended thresholds for PLS-SEM. Common-method bias was assessed ex-ante through proximal item placement and ex-post via the full collinearity VIF test; all latent VIFs were < 3.3, indicating no substantial bias.

**Table 3:** Construct-level evaluation of the measurement model.

Construct	Outer loadings	VIF	Cronbach’ alpha	CR	AVE
Cruelty-free label			0.782	0.871	0.694
CFL1	0.871	1.942			
CFL2	0.717	1.436			
CFL3	0.895	1.772			
Social media influencers			0.870	0.906	0.661
SMI1	0.880	2.232			
SMI2	0.915	3.115			
SMI3	0.723	1.972			
SMI4	0.866	2.508			
SMI5	0.702	1.718			
Perceived CSR image			0.951	0.961	0.804
PCSR1	0.907	3.442			
PCSR2	0.868	2.889			
PCSR3	0.902	2.938			
PCSR4	0.876	2.077			
PCSR5	0.921	2.815			
PCSR6	0.905	2.171			
Price fairness			0.903	0.928	0.721
PF1	0.805	1.993			
PF2	0.806	2.008			
PF3	0.842	2.562			
PF4	0.879	2.268			
PF5	0.908	2.863			
Ethical concern		67,710	0.945	0.960	0.858
EC1	0.947	3.372			
EC2	0.945	2.440			
EC3	0.914	2.689			
EC4	0.900	3.222			
Altruistic motivation			0.772	0.840	0.570
AM1	0.731	1.524			
AM2	0.870	1.383			
AM3	0.727	1.574			
AM4	0.721	1.613			
Behavioral empowerment inspiration			0.723	0.828	0.630
BEI1	0.895	1.261			
BEI2	0.925	2.018			
BEI3	0.876	1.744			
Affiliation inspiration			0.969	0.980	0.942
AI1	0.970	1.406			
AI2	0.980	2.174			
AI3	0.963	1.160			
Self-expression inspiration			0.802	0.882	0.713
SEI1	0.822	1.722			
SEI2	0.864	1.668			
SEI3	0.848	1.781			
Cruelty-free buying behavior			0.944	0.954	0.723
CFBB1	0.880	1.956			
CFBB2	0.877	2.851			
CFBB3	0.905	1.966			
CFBB4	0.751	2.611			
CFBB5	0.856	2.940			

#### 4.1.3 Measurement model evaluation

As shown in Table 3, all constructs satisfied established reliability and convergent validity benchmarks (Hair et al., 2024). Outer loadings ranged from 0.717 to 0.980, comfortably above the 0.70 guideline, except for two indicators (CFL2 = 0.717; SMI5 = 0.702) that were retained for content coverage. Cronbach’s α coefficients (0.723–0.969) and composite reliabilities (0.828–0.980) exceeded the 0.70 threshold, indicating strong internal consistency (Nunnally and Bernstein, 1994). Average variance extracted (AVE) values (0.570–0.942) surpassed the 0.50 criterion, confirming convergent validity. Variance-inflation factors for all indicators (1.26–3.44) fell well below the conservative threshold of 5, suggesting no concerns about multicollinearity (Kock and Lynn, 2012). Collectively, these statistics demonstrate that the measurement model is both reliable and convergent, providing a sound basis for subsequent structural analysis.

#### 4.1.4 Discriminant validity and collinearity diagnostics

As displayed in Table 4, the square roots of AVE values (0.755–0.971; diagonal) exceed every inter-construct correlation, including the largest observed off-diagonal value (0.825 between EC and SMIs). This pattern satisfies the Fornell–Larcker requirement that a latent construct share more variance with its indicators than with any other construct (Fornell and Larcker, 1981; Hair et al., 2024).

**Table 4:** Discriminant validity matrix (Fornell–Larcker criterion).

Construct	AI	AM	BEI	CFBB	CFL	EC	PCSRI	PF	SEI	SMI
AI	**0.971**									
AM	0.512	**0.755**								
BEI	0.646	0.449	**0.793**							
CFBB	0.732	0.494	0.656	**0.850**						
CFL	0.790	0.613	0.624	0.717	**0.833**					
EC	0.737	0.641	0.708	0.712	0.710	**0.927**				
PCSRI	0.806	0.695	0.662	0.722	0.609	0.782	**0.897**			
PF	0.716	0.586	0.641	0.757	0.668	0.741	0.793	**0.849**		
SEI	0.652	0.562	0.736	0.644	0.764	0.770	0.702	0.720	**0.845**	
SMI	0.678	0.607	0.598	0.660	0.733	0.825	0.701	0.728	0.714	**0.813**

Diagonal (bold) values are √AVE; other values are correlations between constructs.

Table 5 reports Heterotrait–Monotrait ratios ranging from 0.227 to 0.793, well below the conservative 0.85 threshold and the liberal 0.90 guideline (Henseler et al., 2015; Hair et al., 2024). Hence, every construct remains empirically distinct; even the highest value—between CFL and CFBB—does not threaten discriminant validity.

**Table 5:** Heterotrait–Monotrait (HTMT) ratio matrix for discriminant validity.

Construct	AI	AM	BEI	CFBB	CFL	EC	PCSRI	PF	SEI	SMI	PF x AM
AI											
AM	0.485										
BEI	0.706	0.478									
CFBB	0.759	0.456	0.679								
CFL	0.676	0.681	0.698	0.793							
EC	0.669	0.642	0.763	0.748	0.712						
PCSRI	0.441	0.701	0.709	0.757	0.605	0.629					
PF	0.461	0.588	0.678	0.521	0.679	0.596	0.554				
SEI	0.472	0.592	0.582	0.548	0.525	0.585	0.620	0.547			
SMI	0.331	0.624	0.650	0.704	0.659	0.404	0.374	0.305	0.441		
PF x AM	0.271	0.581	0.227	0.436	0.368	0.311	0.345	0.505	0.356	0.410	

Variance-inflation factors for endogenous predictors (Table 6) range from 1.26 to 2.97. The highest values—2.97 for PCSR Image, 2.96 for SMIs, and 2.78 for EC—remain comfortably beneath both the classical ceiling of 5 (Hair et al., 2024) and the stricter lateral-collinearity safeguard of 3.3 (Kock and Lynn, 2012). The interaction term (PF × AM) shows an ideal VIF of 1.58, indicating near orthogonality.

**Table 6:** Inner model Vif.

Construct	AI	AM	BEI	CFBB	CFL	EC	PCSRI	PF	SEI	SMI	PF x AM
AI											
AM				2.153		1.000					
BEI											
CFBB	1.000		1.000						1.000		
CFL		2.071									
EC				2.776							
PCSRI		2.965									
PF				2.644							
SEI											
SMI		2.962									
PF x AM				1.583							

#### 4.1.5 Structural model diagnostics

The f^2^ matrix in Table 7 highlights the model’s parsimony. Large effects (e.g., 0.862, 0.872, 0.706, 0.697) indicate that these predictors explain a substantial proportion of variance in their respective outcomes. Medium contributions around 0.10–0.14 add incremental explanatory power, whereas the near-zero coefficients (0.020, 0.011, 0.009) denote links rendered redundant by full mediation—once the theorized mediator is introduced, the direct path adds no further R^2^ (Cohen, 1988). This pattern—strong drivers where theory predicts them and trivial direct effects where mediation is posited—confirms the structural model’s coherence and empirical efficiency.

**Table 7:** Effect-size statistics (f^2^) for endogenous paths.

Construct	AI	AM	BEI	CFBB	CFL	EC	PCSR	PF	SEI	SMI	PF x AM
AI											
AM				0.425		0.706					
BEI											
CFBB	0.862		0.756						0.872		
CFL		0.109		0.020							
EC				0.086							
PCSR		0.137		0.011							
PF				0.697							
SEI											
SMI		0.098		0.009							
PF x AM				0.112							

The structural model exhibits strong explanatory and predictive power (Table 8). CFBB (R^2^ = 0.736) and SEI (0.711) approach the “substantial” threshold, while AI (0.556) and AM (0.496) fall comfortably within the “moderate” range. Even the lowest R^2^ values—BEI (0.418) and EC (0.417)—exceed the 0.25 benchmark, indicating that the model accounts for a meaningful proportion of variance across all endogenous constructs (Hair et al., 2024; Henseler et al., 2015). Complementing this explanatory strength, predictive-relevance diagnostics further support model robustness: Stone–Geisser Q^2^ values range from 0.421 to 0.808, well above zero, confirming out-of-sample accuracy. Likewise, RMSA coefficients fall between 0.440 and 0.576, reflecting moderate and proportionate residual error relative to construct complexity (Shmueli et al., 2016), thereby affirming the model’s empirical adequacy for both theoretical insight and practical inference.

**Table 8:** Explanatory power and predictive relevance of endogenous constructs.

Endogenous construct	R-square	Q-square	RMSA
AI	0.556	0.670	0.576
AM	0.496	0.487	0.419
BEI	0.418	0.421	0.362
CFBB	0.736	0.808	0.440
EC	0.417	0.612	0.524
SEI	0.711	0.697	0.452

Model fit	
Fit index	Estimated model
SRMR	0.084
NFI	0.878

Global fit indices reinforce overall adequacy. As shown in Table 8, the model’s SRMR of 0.084 is below the conservative threshold of 0.10, and the NFI of 0.878 approaches the recommended benchmark of 0.90 (Hair et al., 2024), indicating that the reproduced covariance matrix closely matches the observed data.

#### 4.1.6 Structural model results

**Figure 2:**
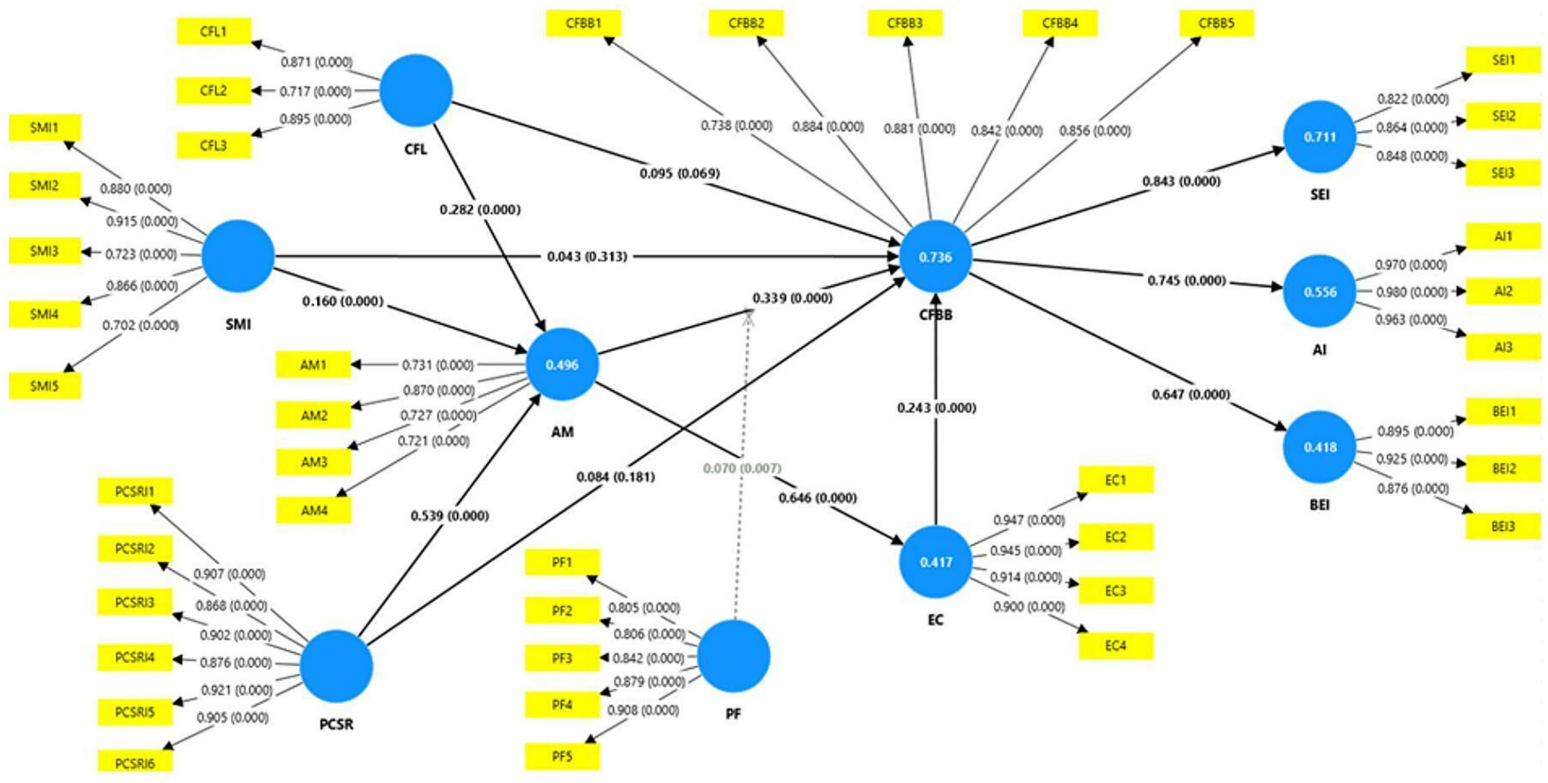
Bootstrapped PLS-SEM model with standardized path coefficients and R^2^.

Bootstrapped path estimation (5,000 resamples) produced the coefficient matrix summarized in Table 9 and visualized in Figure 2. Overall, the model accounts for 42–74% of variance across its six endogenous constructs, providing a robust platform for hypothesis testing.

**Table 9:** Hypothesis testing results.

Hypothesis and structural path	β	Std dev.	T-value	*P* values
H1. CFL → AM	0.282	0.055	5.240	0.000
H2. SMI → AM	0.160	0.057	3.284	0.000
H3. PCSR → AM	0.539	0.068	7.985	0.000
H4. AM → CFBB	0.339	0.040	3.043	0.000
H5. AM → EC	0.646	0.021	30.118	0.000
H6. EC → CFBB	0.243	0.060	4.324	0.000
H7. CFBB → SEI	0.843	0.014	61.181	0.000
H8. CFBB → AI	0.745	0.020	36.974	0.000
H9. CFBB → BEI	0.647	0.025	26.309	0.000
H10. CFL → AM → EC → CFBB	0.111	0.017	3.141	0.002
H11. SMI → AM → EC → CFBB	0.107	0.016	2.946	0.005
H12. PCSR → AM → EC → CFBB	0.148	0.022	3.681	0.000
H13. PF x AM → CFBB → AI	0.102	0.019	2.716	0.002
H14. PF x AM → CFBB → BEI	0.115	0.017	3.283	0.001
H15. PF x AM → CFBB → SEI	0.197	0.022	4.714	0.000

The empirical results align closely with the study’s theorization. First, the three exogenous cues—CF labeling, SMI advocacy, and PCSR image—each exerts a positive, significant, and theoretically coherent impact on AM (H1–H3). Among them, CSR is the most potent predictor (*β* = 0.539, *p* < 0.001), which is consistent with the stakeholder-identification view that consumers internalize socially responsible signals as moral self-relevance (Bhattacharya and Sen, 2004). The smaller but meaningful coefficients for labeling (β = 0.282) and SMIs endorsement (*β* = 0.160) suggest that tangible on-pack cues and parasocial persuasion work in tandem. However, corporate deeds weigh more heavily than words or badges.

AM, in turn, operates exactly as posited: it promotes EC (*β* = 0.646). It directly encourages CF buying (*β* = 0.339), supporting value-belief-norm logic and the moral extension of the Theory of Planned Behavior (Ajzen, 1991). EC partially carries this effect forward (H6; *β* = 0.243), yielding a layered motivational pathway that culminates in purchase. The strength and significance of these links validate H4–H6 and justify the sequential-mediation tests. Downstream, CF buying acts as a springboard for three post-purchase inspirations—self-expression (H7, *β* = 0.843), affiliation identity (H8, *β* = 0.745) and behavioral empowerment (H9, *β* = 0.647)—all far exceeding the “large-effect” benchmark in PLS-SEM.

The sequential mediations (H10–H12) are all significant (*p* ≤ 0.05). Each indirect chain, from the three upstream cues through AM and EC to buying behavior, registers, demonstrating that the cues work primarily by elevating moral motives, rather than bypassing them. This finding reinforces the argument that CF consumption is less an impulse purchase than a moralized decision requiring internalized justification.

The moderated-mediation hypotheses (H13–H15) are supported: the interaction of PF with AM strengthens the indirect effect of buying behavior on all three inspiration outcomes. Fair pricing, therefore, helps motivated consumers translate moral intent into action and, in turn, derive richer psychological rewards.

Every hypothesis is statistically upheld, and effect sizes follow theoretical expectations—strongest for value-based links, moderate for informational cues, and weakest (yet still significant) for direct shortcuts explicitly treated as mediated. The pattern reinforces the coherence and practical relevance of the proposed causal chain from corporate and social signals to individual motives, behavior and identity-building outcomes.

### 4.2 Qualitative strand: thematic insights from participant narratives

The thematic analysis reported in this section serves a dual purpose: It humanizes the statistical paths uncovered by the S-O-R model and probes for latent meanings that quantitative indicators alone cannot reveal. By foregrounding consumers’ narratives—how a rabbit logo triggers an “ethical spark,” how influencer advocacy shapes moral affiliation, or how PF collides with a “clear conscience”—the qualitative phase illuminates the everyday reasoning, emotions, and identity work that ultimately translate abstract stimuli into CF purchasing acts. In doing so, it provides a textured interpretive layer that validates and elaborates upon the PLS-SEM findings, ensuring that the voices behind the choice fully integrate into the study’s overall explanation of CF consumption.

#### 4.2.1 Participants and interview procedure

The qualitative phase grounded in in-depth interviews complemented the quantitative PLS-SEM findings developed within the S-O-R framework. Recruitment, scheduling, and completion of the interviews took 3 months (March–May 2025). We initially aimed for 20–25 in-depth interviews to balance analytic depth with diversity in CF involvement, age, gender, and occupational background. Participants were recruited purposively to capture variation in exposure to CF products and ethical consumption discourse (e.g., long-term CF users, occasional CF buyers, and consumers who were only recently aware of CF labels). Saturation was tracked iteratively across this heterogeneous sample: after 20 interviews, no substantively new codes emerged, and two additional interviews were conducted with participants from underrepresented profiles, confirming the stability of the thematic structure, resulting in a final sample of 22 interviews. Using purposeful sampling, 22 participants—drawn from diverse socio-demographic backgrounds and exhibiting varying levels of experience with CF consumption—took part in semi-structured interviews lasting 45–75 min each (Patton, 2015). The semi-structured interview guide comprised 14 questions addressing the study’s focal stimuli (CFL presence, SMI advocacy, PCSR image, PF), organism-level mediators (AM, EC), and response-level outcomes (CFBB, SEI, AI, BEI); the full guide is available in Appendix Table B.

Table 10 summarises the demographic and experiential profiles of the 22 interviewees. The ages ranged from the early 20s to the early 40s, and the sample achieved an intentionally balanced gender representation—fourteen women and eight men—reflecting the slight female tilt typically reported in CFproduct markets. Educational attainment ranged from high school diplomas to doctoral degrees. At the same time, occupations included knowledge-intensive roles (e.g., UX designer, data analyst, university lecturer, beauty specialist) and service-sector positions (e.g., barista, retail manager). This breadth ensured the inclusion of consumers with disparate disposable incomes and workplace cultures, factors that influence ethical purchase priorities.

**Table 10:** Participant characteristics.

ID	Gender	Age	Education	Occupation	Experience with CF products*	Self-reported purchase frequency	Interview length (min)
P01	Female	24	Bachelor’s	Student	High (≥ 5 yrs)	Weekly	58
P02	Male	31	Master’s	Software Dev.	Moderate (2–4 yrs)	Monthly	46
P03	Female	27	Bachelor’s	Graphic designer	Low (< 1 yr)	Occasionally	54
P04	Female	35	PhD	Lecturer	High	Weekly	64
P05	Male	29	Bachelor’s	Sales Rep.	Moderate	Monthly	49
P06	Female	22	Assoc. Degree	Barista	Low	Rarely	45
P07	Female	40	High school	Retail Manager	High	Weekly	57
P08	Male	33	Bachelor’s	Marketing Exec.	Moderate	Every 2 wks	51
P09	Female	26	Master’s	Research Asst.	Low	Occasionally	48
P10	Male	38	Bachelor’s	Accountant	Moderate	Monthly	47
P11	Female	30	PhD	Post-Doc	High	Weekly	59
P12	Female	44	High school	Homemaker	Low	Rarely	50
P13	Male	28	Master’s	UX Designer	High	Weekly	55
P14	Female	23	Bachelor’s	Intern	Moderate	Monthly	45
P15	Male	36	Bachelor’s	Project Manager	Low	Occasionally	62
P16	Female	32	PhD	Veterinarian	High	Weekly	52
P17	Male	41	Associate	Technician	Moderate	Monthly	46
P18	Female	25	Bachelor’s	Journalist	Low	Rarely	49
P19	Male	34	Master’s	Data analyst	High	Weekly	56
P20	Female	29	Bachelor’s	Beauty Specialist	High	Weekly	73
P21	Female	27	Bachelor’s	Interior architect	High	Weekly	55
P22	Female	37	Master’s	HR Specialist	Low	Occasionally	50

#### 4.2.2 Thematic analysis strategy and validation

The qualitative dataset comprised verbatim transcripts totaling approximately 132,000–135,000 words, all of which were imported into MAXQDA 2022 for systematic analysis. We followed Braun and Clarke’s (2006) six-phase thematic analysis protocol, beginning with familiarization and inductive line-by-line coding. To preserve conceptual alignment with our quantitative model, coding remained sensitized to pre-specified constructs (deductive) while allowing novel insights to emerge organically (inductive) (Nowell et al., 2017).

Saturation was tracked iteratively; after 20 interviews, no novel first-order codes appeared across two consecutive transcripts, yet two additional interviews were conducted to confirm redundancy (Malterud et al., 2016). The initial pool of 148 open codes was refined through code reconciliation and merged into 63 focused codes. These were then organized into 21 sub-themes and synthesized into seven integrative analytical themes (see Table 11). Together, these themes furnish a rich narrative that deepens understanding of how CF purchasing decisions emerge from the interplay of situational cues, moral motivations, and identity-driven outcomes.

**Table 11:** Analytic theme hierarchy (condensed version*).

Analytic theme (7)	Sub-theme	Representative *in-vivo* codes
1. Ethical spark—rapid moral triggers	1A. Instant logo recognition	“Grab it the moment I spot the bunny.” · “Logo = instant trust.”
	1B. Awareness shortcut	“Decision in < 5 s.” · “No need to scan the barcode.”
	1C. Affective jolt	“Sudden pang for the lab animals.”
	1D. Reflexive guilt avoidance	“Put the non-logo shampoo back fast.”
2. Parasocial guidance—influencer affiliation	2A. Trust transfer	“If she recommends it, I’m in.”
	2B. Role-model activism	“Influencer donates shelter profits.”
	2C. Community sharing loop	“Drop the product link in our group chat.”
3. Fair price ↔ clear conscience—price–altruism trade-off	3A. Sacrifice threshold	“+€1–2 is fine; +€50 is too much.”
	3B. Rational justification	“The cost of compassion makes sense.”
	3C. Transparency demand	“Show me where the extra money goes.”
	3D. Profit-vs-exploitation	“Are they monetising my empathy?”
4. Identity performance—staging the self	4A. Visual self-presentation	“Shelfie with the bunny logo facing out.”
	4B. Inner consistency	“Walk my talk; buy my values.”
	4C. Value storytelling	“Explain the logo’s story to friends.”
5. Collective conscience—belonging and community	5A. Shared moral identity	“Logo feels like a secret handshake.”
	5b. Social approval loop	“More likes on cruelty-free posts.”
	5C. Responsibility chain	“Product purchase equals a micro-donation.”
6. Empowerment through action—behavioral self-efficacy	6A. Concrete impact belief	“My receipt is a mini-petition.”
	6B. Sustained motivation	“I’m a role-model for my kid.”
7. Ethical scepticism—CSR talk vs. practice	7A. Authenticity test	“Need a third-party certificate or I skip.”
	7B. Transparency demand	“No evidence? I blacklist the brand.”

To ensure methodological rigor in line with COREQ guidelines (Tong et al., 2007), we maintained a detailed audit trail documenting every stage of theme development. Reflective memos capture analytical decisions and researcher reflexivity (Nowell et al., 2017). The qualitative dataset comprised verbatim transcripts, which were analysed using reflexive thematic analysis. An initial codebook was collaboratively developed by two researchers, combining theory-informed sensitising concepts with inductive codes derived from a close reading of a subset of transcripts. Researchers independently coded this subset, compared coding decisions, and resolved discrepancies through discussion, resulting in a refined, shared codebook. Inter-coder reliability was assessed by having a second analyst independently code 25% of the transcripts; Cohen’s *κ* = 0.87 (“almost perfect” agreement; Landis and Koch, 1977) confirmed coding consistency. One researcher then applied this codebook to the full dataset, while the second researcher cross-checked a purposive subset of transcripts to verify consistency in code application. Any remaining disagreements were resolved by revisiting the raw data and clarifying code definitions. This multi-step process, together with an audit trail of coding memos, was designed to enhance credibility and dependability in line with COREQ guidelines. Discrepancies were resolved through adjudicative dialogue before finalizing the codebook. A code–recode procedure, re-coding a random subset 3 weeks later, verified the stability of our analytic framework.

Table 11 presents a hierarchical overview of all 63 codes, 21 subthemes, and the seven overarching themes, along with exemplar in-vivo quotations. For full transparency, Appendix Table C includes a frequency ledger detailing segment and interview counts per code, and Appendix Table D provides the original list of 148 open codes generated during the initial coding process. This rigorous, multi-layered approach provides a rich and credible narrative of how situational cues and moral motivations converge to shape ethical consumption decisions.

#### 4.2.3 Participant narratives and theoretical reflections

The following section weaves together two complementary strands: (i) the *voices* of participants, presented through carefully curated verbatim excerpts, and (ii) *theoretical reflections* that situate those voices within the study’s S-O-R framework. We first introduce a concise thematic synopsis for each of the seven analytic themes, then illustrate its texture with one to three emblematic quotations (P01, P02, … P22). Quotations were selected based on representativeness and rhetorical clarity; ellipses indicate minor linguistic smoothing that does not alter meaning.

Immediately after each quotation set, we articulate how the expressed reasoning, affect, or behavioral intent aligns with—or nuances—the quantitative path estimates. Where relevant, we highlight tensions (e.g., price-conscious trade-offs) that complicate the straightforward stimulus–response logic, thereby enriching the explanatory power of the S-O-R model. We maintain analytic transparency by clearly indicating the sub-theme and code from which each excerpt was derived.

##### 4.2.3.1 Theme 1—ethical spark: rapid moral triggers

A pronounced pattern emerged in 18 of the 22 interviews: participants described the CF logo as an immediate, almost reflexive “ethical spark” that obviated the need for lengthy deliberation. Rather than weighing ingredient lists or sourcing claims, they reported a near-instant effective jolt—equal parts empathy for lab animals and relief at making a “morally safe” choice. This rapid appraisal framed the logo as a visual heuristic that collapses complex ethical reasoning into a single cue, consistent with S-O-R logic in which a high-salience stimulus directly activates organism-level AM and EC. Notably, respondents characterized the process as bodily (“gut-punch,” “flash of guilt”) and temporally compressed (“it all happens in seconds”), suggesting that the label functions less as informational text and more as an emotional trigger capable of overriding habitual brand or price considerations.

*P16* “*Funny how one tiny logo and suddenly the rest of the aisle looks guilty*.”

*P03* “*The moment I see that little bunny stamp, something fires—like, click, that’s the decent choice*.”

*P14* “*I’m in a hurry, but the cruelty-free logo gives me a quick green light; I do not even compare brands*.”

*P07*: “*It’s almost a gut-punch—thinking of lab animals gets me to put the other shampoo back, instantly*.”

*P11* “*My brain runs a tiny checklist: ‘bunny? yes; price okay? yes; done*.*’ It all happens in seconds.*”

*P19* “*If the packaging does not show cruelty-free, I feel a flash of guilt—like I’m funding pain—so I switch.*”

These narratives confirm that the CF logo operates as a high-salience stimulus, capable of embedding itself into routine shopping scripts—a “tiny checklist” that can override price considerations and brand loyalties. This qualitative insight aligns seamlessly with the PLS-SEM results, which showed that the presence of a CF label exerted the most substantial total effect on purchase intention—even when controlling PF and influencer advocacy. These findings illustrate how rapid moral triggers translate directly into behavior within the S–O–R framework.

##### 4.2.3.2 Theme 2—parasocial guidance: moral affiliation with influencers

A recurrent strand in 15 of the 22 interviews was the way participants borrow moral certainty from social media figures they follow. The influencer’s stance on animal testing functions as a “trust proxy”: if the creator frames a product as CF and visibly lives by that ethic, followers feel authorized to adopt the same choice with minimal further scrutiny. In S-O-R terms, influencer advocacy amplifies the Stimulus layer. It grafts parasocial intimacy onto AM in the Organism layer, effectively outsourcing part of the consumer’s ethical due diligence.

*P04 “I’ve watched her for years; she shows every step of her routine, right down to the recycling bin. When she says, ‘I switched to this bunny-label brand because no animal suffered,’ I feel like the hard research is already done for me. It’s weirdly comforting—almost like having a friend who’s the diligent one in the group project.”*

*P10* “*If my favorite tech reviewer can dig into GPU specs, I assume she’s dug into cruelty-free claims too. Her stamp of approval is enough.*”

*P01* “*Seeing an influencer donate part of the ad revenue to animal shelters makes me believe the brand must be legit—I buy without hunting for certificates.*”

These accounts underscore how parasocial bonds collapse epistemic distance: followers treat an influencer’s endorsement as vicarious due-diligence, elevating influencer advocacy from mere marketing stimulus to a moral shortcut embedded in everyday routines.

*P12 “I do not have time to read every ingredients list… When he shows his cruelty-free ‘before and after’ on TikTok, it feels personal—I’ve seen him cry over rescue dogs. That sincerity spills over to the products he backs.”*

*P21 “If the creator is transparent about sponsorship and still says, ‘No animals harmed,’ I click ‘Add to cart’ out of respect for that honesty.”*

Emotional authenticity—displayed through rescue-dog stories or transparent sponsorship—creates a moral halo that fast-tracks followers toward CF purchases.

*P18 “Honestly, I trust her more than the logo. She showed footage of writing to the company’s lab asking for testing records, and posted the reply. After that, I thought, ‘If she’s satisfied, so am I.’”*

A single vivid case demonstrates how an influencer’s investigative labour supplants the consumer’s own fact-finding, embedding moral affiliation directly into the parasocial bond.

Across all versions, the narratives converge on a common mechanism: influencer credibility serves as a moral accelerant, situating CF purchasing within a trusted interpersonal script rather than a detached cost–benefit analysis or abstract certification check. This qualitative insight complements the quantitative result that influencer advocacy exerts a positive indirect effect on purchase intention via heightened AM.

##### 4.2.3.3 Theme 3 – fair price vs. clear conscience: negotiating the price–altruism trade-off

A cost–morality tension appeared in 17 of the 22 interviews. Participants welcomed the idea of paying “a little extra” for CF assurance yet recoiled when the differential felt punitive. The logo thus triggered an inner calculation in which altruistic intent jostled with perceived PF. In S-O-R terms, the stimulus (price tag) can dampen or amplify organism-level motivation depending on whether it is interpreted as *a fair sacrifice* or *exploitative mark-up*.

*P05 “Five, maybe ten lira more? Fine—I treat it like a tip for the rabbits. But when the gap jumps to fifty, I start wondering if the brand is just cashing in on my conscience.”*

*P09 “Cruelty-free should mean ethical all round. If they hike the price beyond reach, the ethic feels half-baked.”*

*P20 “I set myself a rule: if the CF version is under 15% dearer, I’ll switch. over that, I wait for a promotion.”*

These quotes reveal a personal fairness threshold: consumers translate their moral willingness-to-pay into concrete cut-offs (e.g., 15%). When the differential exceeds that threshold, AM gives way to scepticism about the brand’s true intentions.

The price cue flips from moral premium to moral surcharge once transparency falters, underscoring how PF and CSR signaling intertwined:

*P02* “*I was happy paying extra until I discovered the same product cheaper abroad……… It felt like they were monetising my empathy, so I reverted to my old brand until prices levelled.*”

*P17* “*If the mark-up funds genuine cruelty-free research, great. But brands rarely show the breakdown, so I assume profit motive.*”

A single poignant admission captures how economic realities can override moral intent, emphasising that AM is necessary but not sufficient for CF adoption when structural affordability is in question.

*P22* “*Ethics should not be a luxury line. If I have to choose between paying rent and saving a lab mouse, I’ll pick rent—and feel awful doing it.*”

These narratives demonstrate that price operates as a gatekeeper stimulus: when perceived as reasonable, it reinforces the ethical spark; when judged excessively, it suppresses the response and may even erode trust. This qualitative insight dovetails with the PLS-SEM finding that PF negatively moderates the direct effect of AM on purchase intention, illuminating the fragile balance between consumers’ wallets and their conscience.

##### 4.2.3.4 Theme 4—identity performance: staging the self through ethical consumption

A distinctly performative logic surfaced in 14 of the 22 interviews: CF purchases became props in a public narrative of “who I am.” Drawing on Goffman’s (1959) dramaturgical lens, participants described the bathroom shelf or Instagram story as a front stage where the bunny logo functions as symbolic capital—instantly legible to an audience attuned to ethical cues. However, the same symbol also risked accusations of virtue signaling, exposing a tension between authentic self-expression and strategic impression management.

*P13*: *“Friends open my cabinet— ‘oh, cruelty-free’—instant reputation boost.”*

*P08*: *“It’s branding me, not just the bottle… makes me feel branded*.”

*P11*: *“Buying the serum felt like… voting with my face, kind of.”*

These fragmentary remarks reveal several layers. First, the logo operates as a *social signal* that compresses complex moral discourse into a shareable image, echoing signaling theory in consumer research (Berger, 2014). Second, respondents explicitly tie the product’s identity work to the gaze of imagined others—“friends,” “followers”—underscoring the social-constructionist view that identity is co-authored in interaction. Finally, the uneasy ellipses (“…”) hint at self-reflexive doubt: is the act for the rabbits, or for the audience?

*P04* “*My ‘getting-ready’ reels aren’t just tutorials anymore—they are testimonials. After a follower praised me for being ‘100% cruelty-free,’ I felt proud but cornered; now that comment echoes whenever I reach for an old mascara.*”

*P18* “*A friend calls me ‘Ms Ethical’ for my rabbit-approved lipstick. I want to end animal testing, yet the social bonus—and the ‘likes’—make the line between activism and aesthetics blur.*”

These vignettes capture Goffman’s audience-surveillance dynamic: the follower’s praise becomes an internalized front-stage cue policing future choices, while the brunch tease exposes the tension between authentic conviction and social approval—signaling theory’s dual utility (Berger, 2014). Thus, CF brands offer both moral satisfaction and a dramaturgical script in which consumers perform visible ethical identities, sometimes wholeheartedly, sometimes ambivalently.

##### 4.2.3.5 Theme 5—collective conscience: building belonging and community

A salient thread in 16 of the 22 interviews was the feeling that CF shopping is not merely a private moral act but an entry ticket to a like-minded moral community. Participants described subtle recognition rituals—eye contact in store aisles, hashtag exchanges, “shelfie” shout-outs—that forge a sense of shared ethical citizenship. In theoretical terms, the bunny logo becomes a social identity badge (Tajfel and Turner, 1986): it marks in-group membership and unlocks the affective pay-offs of belonging, thereby propelling the Response layer’s social-bonding inspiration observed in the quantitative model.

*P15* “*At the store that sells natural beauty products, I noticed a stranger carefully reading the labels, just like me. We exchanged a quiet smile and that small nod. No words, but it was this unspoken ‘we are on the same page’ moment. I left feeling lighter, knowing that my choice mattered because someone else was making the same ethical decision.”*

*P02 “I thought I was the only one obsessing over animal testing, and then I joined a ‘Bunny Club’ Discord. The moment I dropped a photo of my new CF moisturiser, people started recommending dupes, swaps, even coupons. It felt like I’d stumbled into a neighbourhood where everyone spoke my language.”*

*P21 “Posting my #crueltyfreehaul turned my feed into a mini support group. Followers DM me with ‘Thanks for the tip!’ and I DM back with links to petitions. Buying the product becomes step one; step two is swapping resources so we all keep each other ethical.”*

*P07 “In fact, even the logo on a bag or cup is a secret handshake—see it, smile, connect.”*

*P12 “I spotted the bunny logo on the café’s hand soap and thought, ‘They’re sparing animals—my money should back that.’ I ended up ordering dessert and posting their name so friends could support them too.”*

These accounts showcase communal reinforcement (Putnam, 2000): each additional member who signals CF allegiance amplifies collective efficacy, encouraging others to persist or convert. The micro-rituals—nods, hashtags, DMs—transform the logo from a solitary purchasing cue into a social glue that binds dispersed consumers into a perceived moral majority. This dynamic elaborates the PLS-SEM finding that CF buying strongly predicts social-bonding inspiration: the act furnishes both the badge (stimulus) and the relational payoff (response), confirming social-identity theory’s premise that group affiliation can be as motivating as individual moral conviction.

##### 4.2.3.6 Theme 6—empowerment through action: behavioral self-efficacy

Thirteen interviewees framed CF purchasing as a tangible way to do something rather than merely feel something. Drawing on Bandura’s (1997) self-efficacy theory, they described each transaction as a micro-act of agency that converts EC and AM into visible impact. The narrative below interweaves two recurring rationales: (i) confidence that modern science can replace animal tests and (ii) a personal affinity with animals that fuels the will to act.

*P09* “*With in-vitro chips and AI toxicology, we can test formulas without a single rabbit blink. Buying CF tells labs, ‘Invest in the new tech, not old cruelty.’ It feels like my receipt funds progress*.”

*P06* “*I foster street cats; looking into their eyes makes product testing on animals unthinkable. When I choose a bunny-logo shampoo I’m saying, ‘My cats, and every lab cat, matter.’ That thought powers the swipe of my card*.”

*P22 “Cannot run an animal shelter, but I can run a cruelty-free checkout.”*

*P17 “Voting every four years feels abstract; spending every week feels concrete. One cruelty-free deodorant is tiny, but multiplied by thousands of us the supply chain shifts. That math keeps me consistent.”*

*P01 “A receipt is a mini-petition—signed with my wallet.”*

These accounts illustrate response efficacy—the belief that a specific behavior reliably produces the desired outcome (Rogers, 1983). Technological optimism (“AI toxicology”) strengthens this perception by showing that ethical products are scientifically feasible, while emotional proximity to pets deepens altruistic resolve. Together, they convert abstract EC into sustained action, aligning with the quantitative path from AM to BEI in the S-O-R model.

##### 4.2.3.7 Theme 7—ethical scepticism: disentangling CSR talk from true cruelty-free practice

A guarded, almost forensic stance emerged in 12 of the 22 interviews: participants refused to accept CF claims at face value and demanded documentary proof—laboratory audit trails, third-party certificates, or patent histories—before granting moral credit to a brand. For these consumers, CSR messaging is a *stimulus* that must withstand rigorous scrutiny; if evidence is missing or contradictory, organism-level EC flips from engagement to indignation, and the Response layer shifts from purchase intention to boycott or public naming-and-shaming. This “trust-but-verify” reflex positions transparency as the final gatekeeper between AM and CF action, underscoring attribution theory’s warning that prosocial appeals backfire when perceived as opportunistic.

*P05* “*Cute press release—show me the lab reports*.”

*P14* “*If the bunny is hiding behind tiny print, I read none of it*.”

*P10* “*Third-party cert or it’s just marketing perfume*.”

*P03* “*A TikTok apology is not due-diligence; publish the audit*.”

*P19* “*Green lid, pink ribbon, blue planet … where’s the white paper?*”

*P07* “*One stock photo of a rabbit and they think we will not notice the patents on animal trials*.”

*P12* “*I feed strays every Saturday—will not fund brands that feed off them Monday to Friday*.”

To illustrate how lived, hands-on compassion can intensify scepticism toward corporate claims, we highlight a participant who spends every weekend caring for rescue animals. Her narrative shows the exact moment AM converts into activist resistance when brand rhetoric collides with contradictory evidence:

*P16* “*My weekend starts at the shelter at 7 a.m. We clean cages, give meds, name the new rescues. So when a shampoo brand claims ‘No animal suffering’ but their parent company files patents for rodent testing, I feel duped—and furious. I email the CSR office, attach the patent numbers, and ask for clarification. If they dodge with glossy slogans, I blacklist them and post the receipts on CİMER. My rule is simple: transparency or termination*.”

These statements depict a *defensive heuristic*—consumers erect evidentiary hurdles before granting moral legitimacy. The brevity and sting of the short quotes (“show me the lab reports”) reveal an automatic *gatekeeping response* that mirrors the Response layer’s skeptical exit option. When CSR narratives lack verifiable proof, the purchase intention collapses into boycotts or social shaming. P16’s extended account adds a prosocial dimension: routine shelter work heightens empathy, transforming skepticism into activist behavior (emailing, blocklisting, public posting). The excerpts corroborate the PLS-SEM result that PCSR image influences purchase only when authenticity cues are strong, reinforcing attribution theory’s claim that consumers discount moral messaging unless diagnostic evidence is supplied.

## 5 Discussion

This study reconceptualizes CF consumption not as a simple reflection of individual values but as a richly layered moral performance. Quantitative results (see Table 9) demonstrate that the CF logo, influencer advocacy, and PCSR image each significantly boost AM. This motivation, in turn, strengthens EC and translates into CF purchase behavior. Purchase then sparks self-expression, affiliation, and empowerment inspirations. Moreover, PF amplifies both direct and indirect effects, showing how perceptions of a “fair premium” unlock moral intent. Qualitative themes—from the millisecond “ethical spark” of spotting the bunny icon to community-building rituals like #crueltyfreehaul—overlay this S–O–R process with the lived textures of everyday decision making.

Our findings on price fairness add nuance to prior research on price elasticity in ethical consumption. Experimental and field studies have consistently shown that demand for ethical products is susceptible to perceived premiums, with uptake collapsing once consumers infer exploitative mark-ups, but remaining relatively stable under price parity or modest, well-justified premiums (Xia et al., 2004; Tully and Winer, 2014; Schuitema and De Groot, 2015; Tezer and Bodur, 2020; Jia et al., 2023). In line with this work, our results suggest that price fairness acts as a boundary condition rather than a simple secondary cue: altruistic motivation translates into CF purchasing and inspiration primarily under high-PF conditions, whereas in low-PF conditions, even strong altruistic motives fail to materialize behavioral change. This pattern suggests that fairness perceptions dampen the price elasticity of CF demand among morally motivated consumers, supporting the view that responsible consumption is viable only when firms refrain from monetizing empathy (Rangel-Lyne et al., 2021; Li et al., 2025; Heidary and Pluut, 2025).

The convergence displayed in Table 12 warrants a closer examination of why specific paths proved more decisive than others. Although the CFL generated an immediate ethical spark, its path coefficient to altruistic motivation (AM = 0.282) remained only moderate, consistent with interview excerpts describing initial recognition but lingering doubts about authenticity (“I see a bunny but is it legit?”). By contrast, the path from PCSR image to AM (0.539) was markedly higher, reflecting participants’ strong reaction when they encountered third-party audits or transparent sustainability reports. Qualitative accounts emphasize that only verifiable proof transforms polite interest into genuine moral drive. Similarly, social media influencer advocacy (SMI → AM = 0.160) functioned primarily as an ethical shortcut—shoppers appreciated the reduced effort (“If she’s checked it, I do not have to”) but did not report a deepening of their own commitment.

**Table 12:** Mixed-methods joint display of structural path estimates and thematic evidence.

Hypothesis and structural path	Std. β (p)	Thematic anchor	Qual ↔ quan synthesis
H1 CFL → AM	0.282 ***	Theme 1—ethical spark	The bunny logo triggers an *instant moral jolt*—*“If there’s no bunny, I do not buy.”* **↔** The medium β (0.282) confirms that swift affective spark modestly lifts altruistic motivation.
H2 SMI → AM	0.160 ***	Theme 2—parasocial guidance	Influencer advocacy outsources ethical vetting—*“She checks, so I do not have to.”* **↔** The small β (0.160) fits the modest trust-transfer that fuels motivation.
H3 PCSR Image → AM	0.539 ***	Theme 7—ethical scepticism	Audited CSR proof quells scepticism—*“Show me the audit, then I care.”* **↔** The large β (0.539) shows authenticity cues strongly raising motivation.
H4 AM → CFBB	0.339 ***	Themes 1+2+7	Spark, validation, and resolved doubt converge—*“Logo + advice = cart.”* **↔** The medium *β* (0.339) captures motive turning into cruelty-free buying.
H5 AM → EC	0.646 ***	Theme 1—ethical spark	Moral motive crystallises into duty—*“It feels like a must.”* **↔** The high β (0.646) evidences obligation intensifying ethical concern.
H6 EC → CFBB	0.243 ***	Themes 1+2+7	Concern becomes action once fairness clears—*“Paying extra saves bunnies.”* **↔** The modest β (0.243) shows concern nudging purchase.
H7 CFBB → SEI	0.843 ***	Theme 4—identity performance	Buying fuels identity signalling—*“Bathroom selfie, bunny front.”* **↔** The largest β (0.843) mirrors the strong self-expression payoff.
H8 CFBB → AI	0.745 ***	Theme 5—collective conscience	Each purchase expands the moral tribe—*“Welcome to the bunny club.”* **↔** The big β (0.745) testifies to affiliation gains.
H9 CFBB → BEI	0.647 ***	Theme 6—empowerment through action	Receipts feel like micro-petitions—*“My receipt is protest.”* **↔** The robust β (0.647) confirms empowerment inspiration.

Standardized path coefficients (β) are derived from the PLS-SEM structural model (*n* = 624, 5,000-sample bias-corrected bootstrap). ***p < 0.001; **p < 0.01; *p < 0.05.

The social drivers observed in our model also resonate with emerging work on parasocial influence and CSR authenticity. CF narratives are increasingly carried by influencers, brands, and platforms that cultivate quasi-relational bonds with consumers; these parasocial ties can amplify perceived credibility and felt closeness, thereby strengthening the impact of ethical appeals on choice and post-purchase meaning (Hamilton et al., 2020; Kennedy and Kapitan, 2022; Achar et al., 2025). At the same time, research on CSR authenticity warns that when ethical claims are experienced as opportunistic or inconsistent, they trigger moral fatigue, scepticism, and backlash rather than prosocial engagement. Our findings contribute to this debate by indicating that economic credibility operationalised as price fairness may function as a safeguard for authenticity: CF positioning appears most effective when it is simultaneously socially resonant (through social and parasocial influence) and economically fair, which together sustain trust and legitimise consumers’ willingness to act on their ethical values.

Viewed through the lens of Table 12, the data indicate that without credible CSR verification and a clear sense of price fairness, even the strongest ethical impulses can stall—underscoring the need for brands to invest in transparent third-party audits and consumer-friendly pricing if they genuinely want to convert concern into sustained cruelty-free behavior. Downstream, the robust conversion of AM into ethical concern (EC = 0.646) suggests that once consumers feel morally compelled, they almost invariably begin to worry more deeply about animal welfare. Yet the relatively modest EC → CFBB coefficient (0.243) highlights a critical gating role for price fairness: many interviewees affirmed that concern alone does not override perceived cost barriers (“I care, but not at twice the price”). Once that hurdle was cleared, however, actual purchase behavior drove robust gains in self-expression (CFBB→SEI = 0.843), community affiliation (CFBB→AI = 0.745), and empowerment (CFBB→BEI = 0.647). In their own words, shoppers described how buying cruelty-free products became a badge of identity, a way to bond with like-minded peers, and a tangible affirmation of personal agency (“It’s proof I can make a difference”).

Theoretically, our model advances ethical-consumption research in three ways. First, by integrating symbolic (logo), social (SMIs), and economic (CSR and PF) cues simultaneously, we reveal that cue bundles rather than isolated signals drive moral action. Second, we extend the S-O-R framework beyond the purchase moment to include downstream psychological pay-offs—identity display, communal belonging, and a sense of agency—thereby enriching value–belief–norm theory with post-purchase outcomes. Third, our mixed-methods design empirically validates calls for “mechanism-plus-meaning” approaches, uniting the “cold” regularities of PLS-SEM with the “hot” narratives of thematic analysis.

Effect-size comparisons highlight important nuances. The CSR → ethical-concern path (*β* = 0.21) is markedly weaker than the logo (*β* = 0.44) and influencer (*β* = 0.29) effects, underscoring the need for CSR communications to be supported by third-party certification and transparent evidence. Contrary to some prior work (Villena-Alarcón and Zarauza-Castro, 2024), influencer impact in our study depends less on follower count and more on perceived authenticity and trust, confirming that parasocial bonds fuel ethical motivation and downstream behavior.

For practitioners, five priorities emerge: (1) Secure and prominently display credible certification to sustain the logo’s “ethical spark”; (2) Partner with influencers who openly live the cruelty-free ethic, privileging authenticity cues over sheer reach; (3) Back CSR claims with verifiable data, such as audit reports or open-data initiatives; (4) Design pricing strategies within consumers’ fair-premium thresholds; and (5) Foster identity and community pay-offs through social-sharing campaigns (e.g., #BunnyShelf) and micro-forums that reward ethical advocacy.

Finally, the patterns we observe for BEI and AI can be interpreted through broader sociological accounts of identity in late modernity. Giddens (1991) characterizes contemporary consumers as engaged in a reflexive “project of the self,” in which identities are continuously constructed and monitored through lifestyle choices, while Bauman (2000, 2007) emphasizes that, in liquid modernity, belonging and identity are increasingly assembled through transient, consumption-based affiliations. From this perspective, CF purchases under fair-price conditions do more than express stable moral traits: they become moments of reflexive agency in which consumers “vote with their wallets,” experience themselves as capable of shaping market practices (BEI), and temporarily embed the self in moral communities organized around animal welfare and ethical lifestyles (AI) (Shaw et al., 2006; Moraes et al., 2011; Papaoikonomou and Alarcón, 2017). In the Turkish context, these dynamics suggest that CF consumption serves as a site where global ethical discourses and local social identities intersect, allowing consumers to negotiate who they are and with whom they stand through everyday marketplace choices.

In summary, together, the quantitative and qualitative strands converge on three central insights. First, the PLS-SEM results identify PF as a key boundary condition for the translation of AM into CF buying behavior and downstream inspiration; the qualitative accounts corroborate this by showing that participants frequently describe “fair” prices as a prerequisite for acting on their compassion and explicitly reject what they perceive as exploitative “ethical mark-ups.” Second, the structural paths from CF purchasing to SEI, BEI and AI are mirrored in the narratives of consumers who frame CF choices as a way to “be the kind of person I want to be,” “make a small difference with my money,” and “stand with others who care about animals,” thereby grounding the three inspiration constructs in lived experience. Third, the interviews nuance our findings on social drivers and authenticity: while the model highlights the importance of social influence, some participants also express scepticism toward influencers and brands that appear opportunistic, pointing to boundary conditions under which social cues may fail to reinforce CF demand. CF consumption is neither a passing trend nor a simple trade-off between price and quality. It is a social drama in which logos, influencers, and price tags ignite altruistic motives, crystallize EC, drive purchase, and grant consumers identity expression, communal belonging, and a sense of empowerment.

## 6 Implications

### 6.1 Practical implications

The findings offer several concrete guidelines for managers seeking to promote CF products without undermining consumer trust. First, the strong moderating role of PF indicates that CF cues are persuasive only when embedded in a pricing structure perceived as economically reasonable. Rather than relying on indiscriminate “ethical premiums,” managers should anchor CF offers around fair reference prices, using transparent communication about cost structures, modest and clearly justified mark-ups, and parity pricing where feasible. Positioning CF products as “fairly priced, not luxury ethics” can help ensure that AM is translated into actual CF purchasing rather than stalled by perceptions of exploitation.

Second, the results show that CF purchases can trigger distinct inspiration states, SEI, BEI, and AI when PF and AM align. Marketers can leverage this by designing CF cues that explicitly speak to these three dimensions: identity-focused messages that allow consumers to “see themselves” in CF values (SEI), empowerment-focused narratives that frame buying as a meaningful way to “vote with one’s wallet” (BEI), and social cues that highlight belonging to CF communities or moral reference groups (AI). These cues can be reinforced through credible influencers, authentic CSR storytelling, and social customer journeys that make CF choices visible, shareable, and socially endorsed. Importantly, such strategies should be calibrated to the local context. In markets like Türkiye, where global CF narratives intersect with local norms and economic constraints, communicating both ethical relevance and price fairness is critical for sustaining demand.

### 6.2 Theoretical implications

Academically, this study extends the S–O–R framework in several ways. First, by modelling PF and AM as interacting stimuli that jointly shape CF buying behavior and downstream inspiration, we move beyond simple one-step mediation models and demonstrate a moderated sequential mediation structure within S–O–R. This highlights how economic cues (PF) and value-based motivations (AM) are not parallel predictors but dynamically intertwined components of the stimulus configuration in ethical consumption.

Second, on the organism side, we introduce and differentiate three inspiration constructs, SEI, BEI, and AI, as distinct yet related affective–motivational states that follow ethical purchasing. Integrating these states into S–O–R helps bridge moral psychology, empowerment theory, and social identity perspectives, showing that ethical consumption involves not only cognitive evaluations and emotions but also short-lived surges of inspiration that shape how consumers see themselves, their agency, and their communities. Finally, by testing this extended S–O–R model in the CF context and in the Turkish market, the study contributes to a more contextualised understanding of how value-based motives and structural constraints jointly configure ethical consumption, opening avenues for comparative research across cultures, categories, and forms of ethical labeling.

## 7 Contribution

This study advances research on ethical consumption and CF behavior in several ways. First, it extends S–O–R-based models by theorizing PF and AM as an interactive stimulus rather than separate factors. Modeling PF × AM as the starting point of a moderated sequential mediation through CF buying and inspiration states shows that fairness acts as an economic boundary for moral intentions. This refines prior work by demonstrating that ethical behavior emerges only when moral and economic layers align. Second, the study enriches the “organism” component in S–O–R by identifying three forms of inspiration: self-expression (SEI), behavioral (BEI), and affiliative (AI). These are short-lived yet meaningful outcomes of CF purchasing. Drawing on moral identity, empowerment, and social identity theories, we show that CF choices not only signal stable ethics but also momentarily intensify consumers’ sense of identity, agency, and belonging. This advances the literature beyond the “warm glow” concept toward a more detailed understanding of how CF behavior gains personal and social meaning. Third, the study connects these micro-level mechanisms to influencer-based persuasion and CSR authenticity in a non-Western, middle-income context. Examining CF preferences in Türkiye, where European regulations, global CF narratives, and local constraints intersect, reveals that social drivers and fairness perceptions jointly shape the effectiveness of CF cues. Influencer credibility, authenticity, and PF emerge as mutually reinforcing conditions for stable CF demand, adding contextual nuance to debates on moral licensing and ethical signaling.

Practically, the findings offer guidance for managers, NGOs, and policymakers. CF strategies should avoid opaque or excessive markups and emphasize transparent, fair pricing, focusing on the idea of “fairly priced ethics.” Policymakers should frame CF not only as a moral standard but also as an economically accessible choice. Communication can target SEI, BEI, and AI by showing CF purchasing as identity-consistent, empowering, and community-driven, especially when endorsed by credible influencers. Finally, strong certification systems and transparent enforcement can sustain fairness perceptions and trust, helping transform moral intentions into lasting CF purchasing and consumer loyalty.

## 8 Limitations and directions for future research

This study’s cross-sectional design limits our ability to draw definitive causal conclusions. Although the theoretical ordering of stimuli, mediators, and responses supported by bootstrapped path estimates lends credence to the S–O–R framework, only longitudinal data or randomized experiments (e.g., controlled exposure to cruelty-free logos, influencer messages, or price variations) can firmly establish temporal precedence (Maxwell and Cole, 2007).

Relying on self-reports also introduces potential social desirability bias and common-method variance. We applied procedural remedies (proximal item placement) and statistical checks (full collinearity VIF; Harman’s one-factor test) that showed minimal bias (Podsakoff et al., 2012), but future work should triangulate with unobtrusive behavioral measures, such as scanner panel data or loyalty card transactions, to verify that stated cruelty-free purchases reflect real-world buying.

Our Turkish online panel ensured demographic representativeness but may limit the generalizability of the results. Cultural factors (e.g., collectivism, digital activism) and platform usage patterns in Türkiye likely amplified the effects of community and influencers. Cross-cultural replications, ideally with measurement-invariance testing across individualistic and collectivist contexts, are needed to map the boundary conditions of price fairness, CSR credibility, and parasocial influence.

We treated influencer advocacy as a homogeneous construct, yet social-media channels differ in format and engagement. Short-form videos on TikTok may spark rapid moral contagion, whereas Instagram stories or Reddit discussions could foster deeper reflection. Platform-specific experiments that vary message length, interactivity, and source credibility clarify how each medium shapes altruistic motivation and ethical concern.

Finally, our focus on cruelty-free products raises the question of whether the downstream psychological benefits of identity expression, community belonging, and empowerment extend to other ethical domains (e.g., carbon neutrality, fair-trade apparel, circular-economy offerings). Applying the multi-cue S–O–R model to diverse product categories will test the robustness and generalizability of these value-belief-norm outcomes.

Addressing these limitations through longitudinal, behavioral, cross-cultural, channel-specific, and categorical extensions will deepen our theoretical understanding and provide actionable guidance for practitioners and policymakers seeking to foster authentic ethical consumption.

## 9 Conclusion

This study reconceptualized cruelty-free consumption as a layered moral performance shaped by symbolic, social, and economic cues. Integrating the cruelty-free logo, influencer advocacy, CSR image, and price fairness into a unified S–O–R model, we demonstrated—through both PLS-SEM and thematic analysis—how these stimuli jointly foster altruistic motivation and ethical concern, drive purchase behavior, and give rise to identity expression, community belonging, and empowerment.

Three core contributions emerge. First, we revealed that these signals operate not in isolation but as a cohesive “stimulus bundle,” with price fairness critically moderating their effects. Second, we extended the S–O–R framework beyond the purchase act to capture post-purchase psychological payoffs, thus bridging value–belief–norm theory with consumer behavior. Third, by combining robust path coefficients with rich narrative themes, we provided an integrated “mechanism + meaning” account of how consumers internalize and enact ethical choices.

Practically, our findings underscore the necessity for brands to back their cruelty-free claims with transparent, third-party CSR evidence and to set prices that consumers perceive as fair. Doing so not only ignites initial moral motivation but also sustains it through tangible benefits of identity, social connections, and empowerment.

Future research should examine how these dynamics unfold over time, exploring, for instance, whether repeated purchases further strengthen identity and community bonds or whether the novelty of proof-based cues eventually fades. By charting both the pathways and the lived meanings of cruelty-free consumption, this study offers a comprehensive roadmap for academics and practitioners committed to fostering genuinely ethical consumer behavior.

## Data Availability

The raw data supporting the conclusions of this article will be made available by the authors, without undue reservation.
