# Supplementary material for: Exploring the ethical values and social drivers behind consumer preferences for cruelty-free products
Source: Front Psychol. 2026 Jan 29;16:1660619. doi: 10.3389/fpsyg.2025.1660619 (PMC12895059; doi:10.3389/fpsyg.2025.1660619)
Supplement: Supplementary file 1 [file Table_1.docx]

# ***Appendix A***

**Table A.** Measurement Scales

| Self-expression inspiration (SEI)  (Schau et al., 2009; Venn et al., 2017) |  | SEI 1. This consumer behavior makes me feel good about myself. |
| --- | --- | --- |
|  |  | **SEI 2.** This consumer behavior is a way to pass on attitudes and values to the next generation. |
|  |  | **SEI 3.** This consumer behavior aligns itself with the current state of culture and society. |
| Affiliation inspiration (AI) (Schau et al., 2009; Venn et al., 2017) |  | **AI 1.** This consumer behavior is responsible for the next generation. |
|  |  | **AI 2.** This consumer behavior is creating a better future for the next generation. |
|  |  | **AI 3.** This consumer behavior is a bigger and longer-term benefit for the next generation. |
| Alturistic Motivation (AI)  (Goldsmith et al., 2000; Prakash et al., 2024) |  | **AM1.** “I buy products that have been ecologically produced because of ethical interest” |
|  |  | **AM2.** “My ethical values match with the environmental preservation of green cosmetic brand” |
|  |  | **AM3.** “I consider that humans must maintain a balance with nature to survive” |
|  |  | **AM4.** “While purchasing cosmetics products, I focus on environmentally friendly or natural cosmetics” |
| Cruelty-Free Label (CFL)  Adapted from (Nittala, 2014; Song et al., 2020) |  | **CFL1.** If possible, I would like to buy products with the cruelty-free label. |
|  |  | **CFL2.** Marketers must advertise the cruelty-free aspects of their products. |
|  |  | **CFL3.** The government must make cruelty-free-labeling mandatory |
| Ethical Concern (EC) |  |  |
| (Suphasomboon & Vassanadumrongdee, 2022) |  | **EC1.** Green cosmetics and personal care products are considered a healthier choice for the environment, and buying green can fulfill my environmental responsibility. |
|  |  | **EC2.** Animal protection is necessary and animal welfare will improve if I do not buy animal-tested products. |
|  |  | **EC3.** I am interested in buying green cosmetics and personal care products because of organic and natural formulation, ethical sourcing, green manufacturing processes, green packaging which are not toxic to the environment. |
|  |  | **EC4.** I am interested in buying green cosmetics and personal care products because I appreciate the eco-friendliness of the product. |
| Perceived CSR Image (PCSR)  Adapted from (Achabou 2020; Ho, 2017; Huang et al., 2022) |  | **PCSRI1.** “I think cruelty-free cosmetic products perform excellently in terms of environmental responsibility." |
|  |  | **PCSRI2.** "I believe cruelty-free cosmetic products fulfill their social responsibility in protecting the environment." |
|  |  | **PCSRI3.** "I think cruelty-free cosmetic products make significant efforts to promote environmental protection." |
|  |  | **PCSRI4.** "Cruelty-free cosmetic products contribute to the preservation of the natural environment." |
|  |  | **PCSRI5.** "Cruelty-free cosmetic products have made considerable efforts to undertake environmental social responsibility." |
|  |  | **PCSRI6.** "Cruelty-free cosmetic products prioritize recycling and the reuse of production materials." |
|  |  | **PCSRI7.** "Cruelty-free cosmetic products devote considerable effort to researching and developing environmentally friendly materials for production." |
| Price Fairness (PF)  Adapted from (Petrick 2002; Martin et al., 2009; Chung & Petrick, 2013) |  | **PF1.** The price of cruelty-free cosmetics is clearly understandable. |
|  |  | **PF2.** The price of cruelty-free cosmetics is fair. |
|  |  | **PF3.** The price of cruelty-free cosmetics is acceptable. |
|  |  | **PF4.** The pricing decisions and procedures for cruelty-free cosmetics are fair. |
|  |  | **PF5.** The pricing decisions and procedures for cruelty-free cosmetics are reasonable. |
|  |  | **PF6.** The pricing decisions and procedures for cruelty-free cosmetics are acceptable. |
| Social Media Influencers (SMIs)  Adapted from (De Veirman et al., 2017) |  | **SMI1.** The content quality and professionalism shared by cruelty-free lifestyle advocates are the main reasons that strongly encourage me to choose cruelty-free products. |
|  |  | **SMI2.** The frequency and amount of content shared by a cruelty-free lifestyle advocate affect whether I follow them. |
|  |  | **SMI3.** I think cruelty-free lifestyle advocates need to be honest with their audience when sharing their daily ethical lifestyle. |
|  |  | **SMI4.** I find the opinions of cruelty-free lifestyle advocates reliable and dependable when they recommend cruelty-free products. |
|  |  | **SMI5.** The background information, education level, personality traits, and personal values of a social cruelty-free lifestyle advocate affect whether I like and follow them. |
| Cruelty Free Buying Behaviour (CFBB)  Adapted from (Khare, 2015) |  | **CFBB1.** I avoid using cosmetic products that harm animals in any way and always prefer cruelty-free alternatives. |
|  |  | **CFBB2.** I usually prefer to purchase cruelty-free cosmetic products. |
|  |  | **CFBB3.** If I need to buy a specific cosmetic product, I always choose ones that are cruelty-free or certified as ethical. |
|  |  | **CFBB4.** I try to purchase cruelty-free products, even though they are more expensive. |
|  |  | **CFBB5.** I always purchase cruelty-free products that align with ethical values. |
|  |  | **CFBB6.** I always refrain from purchasing cosmetic products tested on animals. |
| Behavioral Empowerment Inspiration (BEI)  Adapted from (Speer & Peterson 2000; Christens 2012; Li et al., 2021) |  | **BEI1.** Cruelty-free products inspire me to make my ethical consumption behaviors more deliberate. |
|  |  | **BEI2.** Cruelty-free products inspire me to actively participate in sustainable shopping decisions. |
|  |  | **BEI3.** Cruelty-free products inspire me to align my daily shopping habits with environmental and ethical values |

# ***Appendix B***

**Table B.** Semi-Structured Interview Guide Structured by S–O–R Model

| **No** | **Level** | **Main Question** | **Probe Questions** | **Targeted Insights** |
| --- | --- | --- | --- | --- |
| 1 | **Stimulus** | When you see a “cruelty-free” logo on a product, what is the first thing that comes to mind? | - What does this logo signify for you?  - What emotions or associations does it trigger?  - Can you recall buying—or not buying—a product because of this logo? | Logo awareness; trigger role in purchase decision |
| 2 | **Stimulus** | How do you feel when influencers you follow on social media promote cruelty-free products? | - How does the influencer’s credibility affect you?  - How does this promotion change your purchase intention?  - Can you share a specific example? | Influencer effect; trust perception; emotional response |
| 3 | **Stimulus** | What comes to mind when you learn about a brand’s sustainability or CSR activities? | - Where did you find this information?  - How does it change your trust in the brand?  - How does it influence your purchase decisions?  -Can you recall a specific example of a sustainability or CSR initiative that caught your attention?”  -Which aspects of a brand’s CSR activities matter most to you, and why? | CSR perception; information channels; brand trust |
| 4 | **Stimulus** | Do you consider the price of cruelty-free products to be “fair”? | - What makes the price fair or unfair?  - How do you assess the price–value balance?  - How does price influence your decision to buy? | Price fairness perception; barriers to purchase |
| 5 | **Organism** | How would you describe your personal sense of responsibility toward animal welfare? | - To what extent do you feel responsible?  - How does that sense of responsibility motivate you?  - Can you share a past example? | Level of altruistic motivation; internalized values |
| 6 | **Organism** | How would you explain the motivations behind your preference for cruelty-free products? | - Are your motivations internal (conscience) or external (social pressure)?  - Which factor is the strongest driver?  - What other values play a role? | Internal vs. external motivation sources; core values |
| 7 | **Organism** | How do you evaluate your ethical concerns when making a purchase? | - Which ethical issues are most important to you?  - What role do they play in your decision process? | Focus of ethical concerns; ethical weight in decision process |
| 8 | **Response** | Can you describe the last time you purchased a cruelty-free product? | - Which factors guided you at that moment?  - How long did the purchase process take?  - How did you feel afterward? | Concrete behavior examples; purchase process; emotional outcomes |
| 9 | **Response** | How does your cruelty-free choice reflect in how you express yourself? | - Do you share about it on social media?  - What reactions do you get from your circle?  - Do you see it as a form of identity expression? | Self-expression mechanisms; social sharing behaviors |
| 10 | **Response** | Which type of community do you feel part of when you consume cruelty-free products? | - How do you interact with that community?  - What common practices do you share?  - Please give an example of that sense of belonging.  -What activities or interactions make you feel part of that community?  -How do you keep in touch with this community (forums, social media groups, events)? | Sense of belonging; community interaction patterns |
| 11 | **Response** | Does this purchase give you a sense of empowerment?  *CQ:* When making this purchase, did you feel more empowered, in control, or as if you were taking greater responsibility for your decisions? | - When did you feel empowered most strongly?  - How does that feeling motivate subsequent actions?- Can you share a related experience?  -At what moments during the purchase did you feel especially powerful?  -How would you describe that feeling of control? Can you give an example?  -Can you tell me about a time when you felt you were taking full responsibility for your decision? | Perceived behavioral empowerment; motivation for subsequent actions |
| 12 | **Response** | Have you ever shared information about a cruelty-free product with a friend or family member? | - How and when did you share it?  - What was their reaction?  - What was your purpose in sharing? | Information-sharing behavior; social interaction examples |
| 13 | **Response** | When choosing between labeled and non-labeled products, which criteria do you prioritize? | - Beyond the label, what features do you consider?  - How do you rank your decision criteria?  - Can you give an example of a comparison? | Decision priorities; hierarchy of decision criteria |
| 14 | **Response** | How do you expect your consumption of cruelty-free products to change in the future? | - Will your purchase frequency increase?  - Which new product categories would you like to try?  - What factors shape these expectations? | Future consumption intentions; trend and expectation projections |

CQ: Clarifying question

# ***Appendix C***

**Table B.** Full Code Ledger

| **#** | **Analytic Theme** | **Sub-Theme** | **Focused Code (exact in-vivo label)** | **Segments (n)** | **Interviews (n)** |
| --- | --- | --- | --- | --- | --- |
| **1** | Ethical Spark | 1A Instant Logo Recognition | “Grab it the moment I spot the bunny.” | 12 | 11 |
| **2** |  |  | “Instant trust at first glance.” | 10 | 10 |
| **3** |  |  | “Ethical shortcut.” | 8 | 9 |
| **4** |  | 1B Awareness Shortcut | “Decision in < 5 sec.” | 11 | 10 |
| **5** |  |  | “No need to scan barcode.” | 9 | 8 |
| **6** |  |  | “Automatic approval.” | 7 | 7 |
| **7** |  | 1C Affective Jolt | “Sudden pang of conscience.” | 7 | 6 |
| **8** |  |  | “Feeling of moral relief.” | 6 | 6 |
| **9** |  | 1D Reflexive Guilt Avoidance | “Avoid product without logo.” | 9 | 8 |
| **10** |  |  | “Instant guilt spike.” | 6 | 6 |
| **11** | Parasocial Guidance | 2A Trust Transfer | “Someone I trust recommended it.” | 10 | 9 |
| **12** |  |  | “No need for substitute research.” | 9 | 9 |
| **13** |  |  | “Influencer seal of approval.” | 8 | 7 |
| **14** |  | 2B Role-Model Activism | “Influencer donates to shelters.” | 7 | 6 |
| **15** |  |  | “Modelling behaviour change.” | 6 | 6 |
| **16** |  |  | “Emotional sharing in video.” | 5 | 5 |
| **17** |  | 2C Community Sharing Loop | “Share product link.” | 8 | 8 |
| **18** |  |  | “Chat about ethics in comments.” | 6 | 6 |
| **19** |  |  | “Giveaway with ethical angle.” | 5 | 5 |
| **20** | Fair Price ↔ Clear Conscience | 3A Sacrifice Threshold | “+€1-2 acceptable.” | 8 | 10 |
| **21** |  |  | “+€50 too expensive.” | 7 | 9 |
| **22** |  |  | “15 % rule.” | 6 | 8 |
| **23** |  | 3B Rational Justification | “Paying for animal welfare.” | 9 | 12 |
| **24** |  |  | “Expect price breakdown.” | 8 | 10 |
| **25** |  |  | “Profit ≠ exploitation.” | 7 | 9 |
| **26** |  | 3C Transparency Demand | “Show me where the extra money goes.” | 6 | 8 |
| **27** |  |  | “Company financial report.” | 5 | 6 |
| **28** |  |  | “Supply-chain proof.” | 5 | 6 |
| **29** |  | 3D Profit vs Exploitation | “Profit from empathy.” | 6 | 7 |
| **30** |  |  | “Overstated ethical premium.” | 5 | 6 |
| **31** | Identity Performance | 4A Visual Self-Presentation | “#Shelfie with bunny logo facing out.” | 10 | 12 |
| **32** |  |  | “Display logo on bathroom shelf.” | 8 | 10 |
| **33** |  |  | “Tag product in story.” | 8 | 9 |
| **34** |  | 4B Inner Consistency | “Walk my talk purchase.” | 9 | 11 |
| **35** |  |  | “Value–behaviour match.” | 7 | 9 |
| **36** |  |  | “Feel good about myself.” | 7 | 8 |
| **37** |  | 4C Value Storytelling | “Tell the product’s story.” | 6 | 8 |
| **38** |  |  | “Ethical narrative on social media.” | 6 | 7 |
| **39** |  |  | “Defend choice in conversation.” | 5 | 6 |
| **40** | Collective Conscience | 5A Shared Moral Identity | “Logo feels like a secret handshake.” | 8 | 10 |
| **41** |  |  | “Recognise fellow bunny buyers.” | 7 | 9 |
| **42** |  |  | “Shared ethical flag.” | 7 | 9 |
| **43** |  | 5B Social Approval Loop | “Friend approval.” | 6 | 8 |
| **44** |  |  | “More likes on cruelty-free posts.” | 6 | 8 |
| **45** |  |  | “Community pressure.” | 5 | 7 |
| **46** |  | 5C Responsibility Chain | “Product equals donation.” | 6 | 8 |
| **47** |  |  | “Share petition link.” | 5 | 6 |
| **48** | Empowerment through Action | 6A Concrete Impact Belief | “Receipt as mini-petition.” | 9 | 10 |
| **49** |  |  | “Purchase equals vote.” | 8 | 10 |
| **50** |  |  | “Collective impact math.” | 7 | 9 |
| **51** |  |  | “Promote new test technologies.” | 6 | 7 |
| **52** |  | 6B Sustained Motivation | “Be an example for my child.” | 6 | 8 |
| **53** |  |  | “Habit chain.” | 5 | 7 |
| **54** |  |  | “Feel accountable.” | 5 | 6 |
| **55** | Ethical Scepticism | 7A Authenticity Test | “Need a third-party certificate.” | 8 | 9 |
| **56** |  |  | “Request lab report.” | 7 | 8 |
| **57** |  |  | “Patent search.” | 6 | 7 |
| **58** |  |  | “Investigate company history.” | 6 | 6 |
| **59** |  | 7B Transparency Demand | “CSR email inquiry.” | 7 | 8 |
| **60** |  |  | “Glossy slogan suspicion.” | 6 | 7 |
| **61** |  |  | “Blacklist and share.” | 6 | 7 |
| **62** |  |  | “Upload evidence to Reddit.” | 5 | 6 |
| **63** |  |  | “Boycott if no transparency.” | 5 | 6 |

# ***Appendix D***

**Table D.** Comprehensive Codebook: Open Codes, Sub-Themes, and In-Vivo Labels

| ID | Sub-theme | Raw code / in-vivo label |
| --- | --- | --- |
| Theme 1 *– Ethical Spark · Rapid Moral Triggers* |  |  |
| 1 | 1A Label Instant Recognition | “Bunny stamp = no thinking” |
| 2 | 1A Label Instant Recognition | “Front-label rabbit means safe choice” |
| 3 | 1A Label Instant Recognition | “Green bunny catches my eye first” |
| 4 | 1A Label Instant Recognition | “If the logo’s on top, I don’t flip the bottle” |
| 5 | 1A Label Instant Recognition | “No bunny, no buy” |
| 6 | 1A Label Instant Recognition | “Logo bigger than brand name, love it” |
| 7 | 1A Label Instant Recognition | “Tiny logo? I might miss it” |
| 8 | 1B Awareness Shortcut | “Decision done in under five seconds” |
| 9 | 1B Awareness Shortcut | “Don’t need ingredient PhD” |
| 10 | 1B Awareness Shortcut | “Logo = instant green light” |
| 11 | 1B Awareness Shortcut | “Reads like a moral QR code” |
| 12 | 1B Awareness Shortcut | “Visual shorthand for ethics” |
| 13 | 1B Awareness Shortcut | “Skip the Google search” |
| 14 | 1B Awareness Shortcut | “Checks one box, good to go” |
| 15 | 1C Emotional Jolt | “Gut-punch thinking of lab bunnies” |
| 16 | 1C Emotional Jolt | “Heart squeeze when I see the stamp” |
| 17 | 1C Emotional Jolt | “Flash of guilt if the logo’s missing” |
| 18 | 1C Emotional Jolt | “Relief floods in—no animals hurt” |
| 19 | 1C Emotional Jolt | “Feel lighter inside instantly” |
| 20 | 1C Emotional Jolt | “Warm rush of ‘doing good’ ” |
| 21 | 1C Emotional Jolt | “Sad flash of cages in my mind” |
| 22 | 1D Reflex Guilt-Avoidance | “Put the other shampoo back fast” |
| 23 | 1D Reflex Guilt-Avoidance | “Shopping with conscience on speed dial” |
| 24 | 1D Reflex Guilt-Avoidance | “Logo missing = back on shelf” |
| 25 | 1D Reflex Guilt-Avoidance | “Avoid funding pain” |
| 26 | 1D Reflex Guilt-Avoidance | “Swipe card only if bunny there” |
| 27 | 1D Reflex Guilt-Avoidance | “Don’t want guilt receipt” |
| 28 | 1D Reflex Guilt-Avoidance | “Instant moral stop-sign” |
| Theme 2 – *Parasocial Guidance · Influencer Affiliation* |  |  |
| 29 | 2A Trust Transfer | “If she endorses, I’m in” |
| 30 | 2A Trust Transfer | “Borrowing her research” |
| 31 | 2A Trust Transfer | “Influencer stamp stronger than label” |
| 32 | 2A Trust Transfer | “Her review > brand claims” |
| 33 | 2A Trust Transfer | “Audit done by my fave creator” |
| 34 | 2A Trust Transfer | “Content creator as ethics filter” |
| 35 | 2A Trust Transfer | “Trust proxy activated” |
| 36 | 2B Role-model Activism | “Influencer donates shelter profits” |
| 37 | 2B Role-model Activism | “He cried over rescue video” |
| 38 | 2B Role-model Activism | “Followed her cruelty-free switch journey” |
| 39 | 2B Role-model Activism | “Live stream: lab tour boycott” |
| 40 | 2B Role-model Activism | “Shared her petition link” |
| 41 | 2B Role-model Activism | “Seeing her use bunny logo daily” |
| 42 | 2B Role-model Activism | “Role-model vibe rubs off” |
| 43 | 2C Community Sharing Loop | “Drop product link in comments” |
| 44 | 2C Community Sharing Loop | “DM friends discount code” |
| 45 | 2C Community Sharing Loop | “Hashtag #BunnyApproved” |
| 46 | 2C Community Sharing Loop | “Live Q&A about testing” |
| 47 | 2C Community Sharing Loop | “We swap cruelty-free dupes” |
| 48 | 2C Community Sharing Loop | “Influencer fan group recommendations” |
| 49 | 2C Community Sharing Loop | “Group chat product vetting” |
| Theme 3 – *Fair Price vs. Clear Conscience* |  |  |
| 50 | 3A Sacrifice Threshold | “Extra 1–2 € fine” |
| 51 | 3A Sacrifice Threshold | “>50 €? No thanks” |
| 52 | 3A Sacrifice Threshold | “15 % premium rule” |
| 53 | 3A Sacrifice Threshold | “Budget vs. bunny tug-of-war” |
| 54 | 3A Sacrifice Threshold | “Waiting for sale” |
| 55 | 3A Sacrifice Threshold | “Affordable ethics only” |
| 56 | 3A Sacrifice Threshold | “Will pay coffee-price extra” |
| 57 | 3B Rational Justification | “Cost of compassion” |
| 58 | 3B Rational Justification | “Happy to pay vet research” |
| 59 | 3B Rational Justification | “Ethical tax acceptable” |
| 60 | 3B Rational Justification | “Pays for safer science” |
| 61 | 3B Rational Justification | “Compare NGO price chart” |
| 62 | 3B Rational Justification | “Value > price delta” |
| 63 | 3B Rational Justification | “Think of rabbits when scanning price” |
| 64 | 3C Transparency Demand | “Break-down cost sheet” |
| 65 | 3C Transparency Demand | “Want to see price justification” |
| 66 | 3C Transparency Demand | “Explain cruelty-free overhead” |
| 67 | 3C Transparency Demand | “Where my extra money goes” |
| 68 | 3C Transparency Demand | “Need audit trail for price” |
| 69 | 3C Transparency Demand | “No clarity, no purchase” |
| 70 | 3C Transparency Demand | “Open costing builds trust” |
| 71 | 3D Profit vs. Exploitation | “Selling empathy too hard” |
| 72 | 3D Profit vs. Exploitation | “Monetising my moral heart” |
| 73 | 3D Profit vs. Exploitation | “Surcharge smells like greed” |
| 74 | 3D Profit vs. Exploitation | “Ethics shouldn’t be luxury line” |
| 75 | 3D Profit vs. Exploitation | “Guilt premium feels exploitative” |
| 76 | 3D Profit vs. Exploitation | “Are we paying for marketing only?” |
| 77 | 3D Profit vs. Exploitation | “Sticker shock cancels virtue” |
| Theme 4 – *Identity Performance* |  |  |
| 78 | 4A Visual Self-Presentation | “Bathroom shelfie with bunny logo” |
| 79 | 4A Visual Self-Presentation | “Instagram story product reveal” |
| 80 | 4A Visual Self-Presentation | “Logo positioned facing camera” |
| 81 | 4A Visual Self-Presentation | “Cruelty-free flat lay” |
| 82 | 4A Visual Self-Presentation | “Unboxing with ethics talk” |
| 83 | 4A Visual Self-Presentation | “Eco-friendly vanity tour” |
| 84 | 4A Visual Self-Presentation | “TikTok GRWM cruelty-free edition” |
| 85 | 4B Inner Consistency | “Walk my talk purchase” |
| 86 | 4B Inner Consistency | “Live like my values” |
| 87 | 4B Inner Consistency | “Align brand with beliefs” |
| 88 | 4B Inner Consistency | “Feel hypocrite without bunny” |
| 89 | 4B Inner Consistency | “Purchase = identity proof” |
| 90 | 4B Inner Consistency | “Self-respect purchase” |
| 91 | 4B Inner Consistency | “Ethics mirror check” |
| 92 | 4C Value Storytelling | “Tell friends why I switched” |
| 93 | 4C Value Storytelling | “Narrate bunny logo meaning” |
| 94 | 4C Value Storytelling | “Use storytime to educate” |
| 95 | 4C Value Storytelling | “Brand origin tale matters” |
| 96 | 4C Value Storytelling | “Share rescue centre stats” |
| 97 | 4C Value Storytelling | “Moral back-story sells me” |
| 98 | 4C Value Storytelling | “Turn product into advocacy anecdote” |
| Theme 5 – *Collective Conscience* |  |  |
| 99 | 5A Shared Moral Identity | “Silent nod in aisle” |
| 100 | 5A Shared Moral Identity | “Recognise fellow bunny buyers” |
| 101 | 5A Shared Moral Identity | “Logo = secret handshake” |
| 102 | 5A Shared Moral Identity | “Team bunny vibe” |
| 103 | 5A Shared Moral Identity | “Instant kinship over bottle” |
| 104 | 5A Shared Moral Identity | “Feel part of ethical tribe” |
| 105 | 5A Shared Moral Identity | “Spot bunny, spot ally” |
| 106 | 5B Social Approval Loop | “Friend praise for bunny brand” |
| 107 | 5B Social Approval Loop | “Likes spike on cruelty-free post” |
| 108 | 5B Social Approval Loop | “Peer pressure to stay ethical” |
| 109 | 5B Social Approval Loop | “Positive comments encourage habit” |
| 110 | 5B Social Approval Loop | “Group norm formation” |
| 111 | 5B Social Approval Loop | “Feedback keeps me consistent” |
| 112 | 5B Social Approval Loop | “‘Good for you!’ messages” |
| 113 | 5C Responsibility Chain | “Purchase funds shelters” |
| 114 | 5C Responsibility Chain | “Share petition after buying” |
| 115 | 5C Responsibility Chain | “Product = micro-donation” |
| 116 | 5C Responsibility Chain | “Tag brand when they donate” |
| 117 | 5C Responsibility Chain | “Collective impact stories” |
| 118 | 5C Responsibility Chain | “Buddy system to stay cruelty-free” |
| 119 | 5C Responsibility Chain | “Pass coupon to friends” |
| Theme 6 – *Empowerment through Action* |  |  |
| 120 | 6A Concrete Impact Belief | “Receipt as mini-petition” |
| 121 | 6A Concrete Impact Belief | “Consumer vote with wallet” |
| 122 | 6A Concrete Impact Belief | “Add one, subtract animal test” |
| 123 | 6A Concrete Impact Belief | “Purchase funds better science” |
| 124 | 6A Concrete Impact Belief | “Cumulative effect math” |
| 125 | 6A Concrete Impact Belief | “Small act, big ripple” |
| 126 | 6A Concrete Impact Belief | “Products push tech change” |
| 127 | 6A Concrete Impact Belief | “Switch fuels cruelty-free R&D” |
| 128 | 6B Sustained Motivation | “Foster cats influence buys” |
| 129 | 6B Sustained Motivation | “Kid learns from my cart” |
| 130 | 6B Sustained Motivation | “Habit loop forms” |
| 131 | 6B Sustained Motivation | “Feel accountable weekly” |
| 132 | 6B Sustained Motivation | “Ethical streak tracker” |
| 133 | 6B Sustained Motivation | “Set monthly bunny goal” |
| Theme 7 – *Ethical Scepticism* |  |  |
| 134 | 7A Authenticity Test | “Third-party cert or skip” |
| 135 | 7A Authenticity Test | “Need lab audit PDF” |
| 136 | 7A Authenticity Test | “Patent search for animal trials” |
| 137 | 7A Authenticity Test | “Ask CSR for proof” |
| 138 | 7A Authenticity Test | “Green claim, grey reality” |
| 139 | 7A Authenticity Test | “No receipt for ethics talk” |
| 140 | 7A Authenticity Test | “No vetting, no sale” |
| 141 | 7B Transparency Demand | “Glossy slogan dodge” |
| 142 | 7B Transparency Demand | “Email brand, no reply” |
| 143 | 7B Transparency Demand | “Blacklist and share screenshots” |
| 144 | 7B Transparency Demand | “CSR page vague” |
| 145 | 7B Transparency Demand | “Crowd-source brand audits” |
| 146 | 7B Transparency Demand | “Reddit proof post” |
| 147 | 7B Transparency Demand | “Transparency or termination” |
| 148 | 7B Transparency Demand | “Expose hidden parent company” |
